# Spatial Distribution of Carbon Stored in Forests of the Democratic Republic of Congo

**DOI:** 10.1038/s41598-017-15050-z

**Published:** 2017-11-08

**Authors:** Liang Xu, Sassan S. Saatchi, Aurélie Shapiro, Victoria Meyer, Antonio Ferraz, Yan Yang, Jean-Francois Bastin, Norman Banks, Pascal Boeckx, Hans Verbeeck, Simon L. Lewis, Elvis Tshibasu Muanza, Eddy Bongwele, Francois Kayembe, Daudet Mbenza, Laurent Kalau, Franck Mukendi, Francis Ilunga, Daniel Ebuta

**Affiliations:** 1 Institute of Environment and Sustainability, University of California, Los Angeles, CA USA; 2 Jet Propulsion Laboratory, California Institute of Technology, Pasadena, CA USA; 3 World Wide Fund for Nature(WWF) Germany Biodiversity Unit, Berlin, Germany; 4 Landscape Ecology and Plant Production Systems Unit, Université libre de Bruxelles, Bruxelles, Belgium; 5 BIOSE department, Gembloux Agro Bio Tech, Gembloux, Belgium; 6 Southern Mapping Company, Airborne LiDAR Survey Unit, Johannesburg, South Africa; 7 Isotope Bioscience Laboratory - ISOFYS, Ghent University, Ghent, Belgium; 8 CAVElab – Computational and Applied Vegetation Ecology, Ghent University, Ghent, Belgium; 9 School of Geography, University of Leeds, Leeds, UK; 10 Department of Geography, University College London, London, UK; 11 World Wide Fund for Nature (WWF), Kinshasa, Democratic Republic of the Congo; 12 Observatoire Satellital des Forets d’Afrique Central (OSFAC), Kinshasa, Democratic Republic of the Congo; 13 Direction des Inventaires et Aménagement Forestiers (DIAF), Kinshasa, Democratic Republic of the Congo

## Abstract

National forest inventories in tropical regions are sparse and have large uncertainty in capturing the physiographical variations of forest carbon across landscapes. Here, we produce for the first time the spatial patterns of carbon stored in forests of Democratic Republic of Congo (DRC) by using airborne LiDAR inventory of more than 432,000 ha of forests based on a designed probability sampling methodology. The LiDAR mean top canopy height measurements were trained to develop an unbiased carbon estimator by using 92 1-ha ground plots distributed across key forest types in DRC. LiDAR samples provided estimates of mean and uncertainty of aboveground carbon density at provincial scales and were combined with optical and radar satellite imagery in a machine learning algorithm to map forest height and carbon density over the entire country. By using the forest definition of DRC, we found a total of 23.3 ± 1.6 GtC carbon with a mean carbon density of 140 ± 9 MgC ha^−1^ in the aboveground and belowground live trees. The probability based LiDAR samples capture variations of structure and carbon across edaphic and climate conditions, and provide an alternative approach to national ground inventory for efficient and precise assessment of forest carbon resources for emission reduction (ER) programs.

## Introduction

Tropical forests provide valuable ecosystem services, notably by storing vast amounts of carbon in biomass and serving as an important reservoir for climate change mitigation^1^. Since the 2009 United Nations Framework Convention on Climate Change (UNFCCC)^2^, establishing robust and transparent national forest monitoring systems has become a key policy incentive for reporting estimates of forest area and carbon stocks and developing infrastructure to reduce emissions from deforestation and degradation (REDD+)^3^. In a national REDD+ policy framework, a forest reference emission level (expressed as tons of CO_2_ equivalent per year) must be set, and future emissions must be evaluated against the reference level as part of a monitoring (or measuring), reporting and verification (MRV) system to determine whether a country has or has not made significant emission reductions in the land use sector^4^. The uncertainty around reference emission levels and the resulting emissions from activity data such as land use and land use change in forest (LULUCF) must also be quantified. Because of the principle of conservativeness, results from the use of the lower uncertainty bounds for emission factors (as the difference of carbon stocks resulting from land use change) for the reference scenario must be adopted in order to avoid over-crediting future reductions. Meeting these conditions for national or regional scale REDD+ programs require accurate inventory of forest carbon stocks and changes that capture regional variability of forest aboveground biomass and land use patterns^5,6^.

Many important technical and political questions remain to be answered regarding how REDD+ emission reduction projects and programs will be implemented at the national level. Smaller voluntary-sector projects have been operating in many countries across the tropics since 2006 under the Verified Carbon Standard (VCS) and Carbon Communities, & Biodiversity Alliance (CCBA) Standards, amongst others, and provide much guidance as to how national-level schemes could operate. However, at the national level, emission or removal estimates from land use and cover (LULC) change require information on both the area of forest change and the corresponding carbon stocks of the ecosystems that are deforested. Such information is either not available or highly uncertain in many countries with extensive tropical forests. Much of the successful applications on emissions of tropical forests to date are the areal estimates of deforestation; yet significant uncertainty exist in forest carbon stocks and emission factors, particularly when considering jurisdictional and national level emissions^7,8^.

Currently, carbon stock estimates associated with reference level in tropical countries are often based on a small number of forest plots in intensive sites without any systematic design, paired with remote sensing methods (satellite or aerial)^9^. There is a general consensus in the scientific literature that satellite imagery can provide monitoring tools for forest cover change over time at national and local scales^10,11^. While methods to map carbon stocks directly from satellite remote sensing observations have not been perfected nor made operational yet – current effort and practices often use a combination of airborne and satellite imagery, trained by plot-level field measurements at the national scale^12–14^.

The most advanced remote sensing methodologies for estimating and mapping carbon stocks rely strongly on Light Detection And Ranging (LiDAR) observations of forest structure that can be readily converted to aboveground biomass (AGB) and extrapolated over the landscape using satellite imagery^9,15,16^. There are, however, uncertainty associated with this methodology due to the uneven or nonrandom LiDAR sampling of study region, poor training of LiDAR data to forest biomass, and sensitivity of the satellite imagery to vegetation structure and biomass when extrapolating LiDAR biomass estimations over the landscape^17,18^.

Here we develop, for the first time, a national level forest carbon map in a tropical country based on probability sampling of forest structure and biomass by airborne LiDAR data. We focus on the Democratic Republic of Congo (DRC), which holds the second largest extent of tropical forests after Brazil and develop an unbiased estimator of AGB using LiDAR samples trained with ground inventory plots. The LiDAR AGB estimates are integrated with the geospatial modeling based on the Maximum Entropy (ME) machine learning algorithm to produce a biomass map at 100 m (1 ha) resolution of the entire DRC. Our overall methodology is designed to reduce errors from different sources and provide uncertainty estimates at the 1-ha pixel scales that can be readily verified using inventory plots. The AGB map along with the uncertainty can provide emission factors for land use and land cover activities at national and subnational scales. The approach is analogous to a designed-based inference with hierarchical modeling^19,20^ consisting of three steps: (1) ground plots used as the first source of information, providing samples of structure and biomass; (2) airborne LiDAR scanning (ALS) data sampled across the country used as the second source of information and a proxy for national level forest inventory, and (3) geospatial modeling and satellite imagery used as the third source of information for wall-to-wall mapping of forest biomass and carbon stocks.

## Methods

### LiDAR Sampling Design

**Figure 1:**
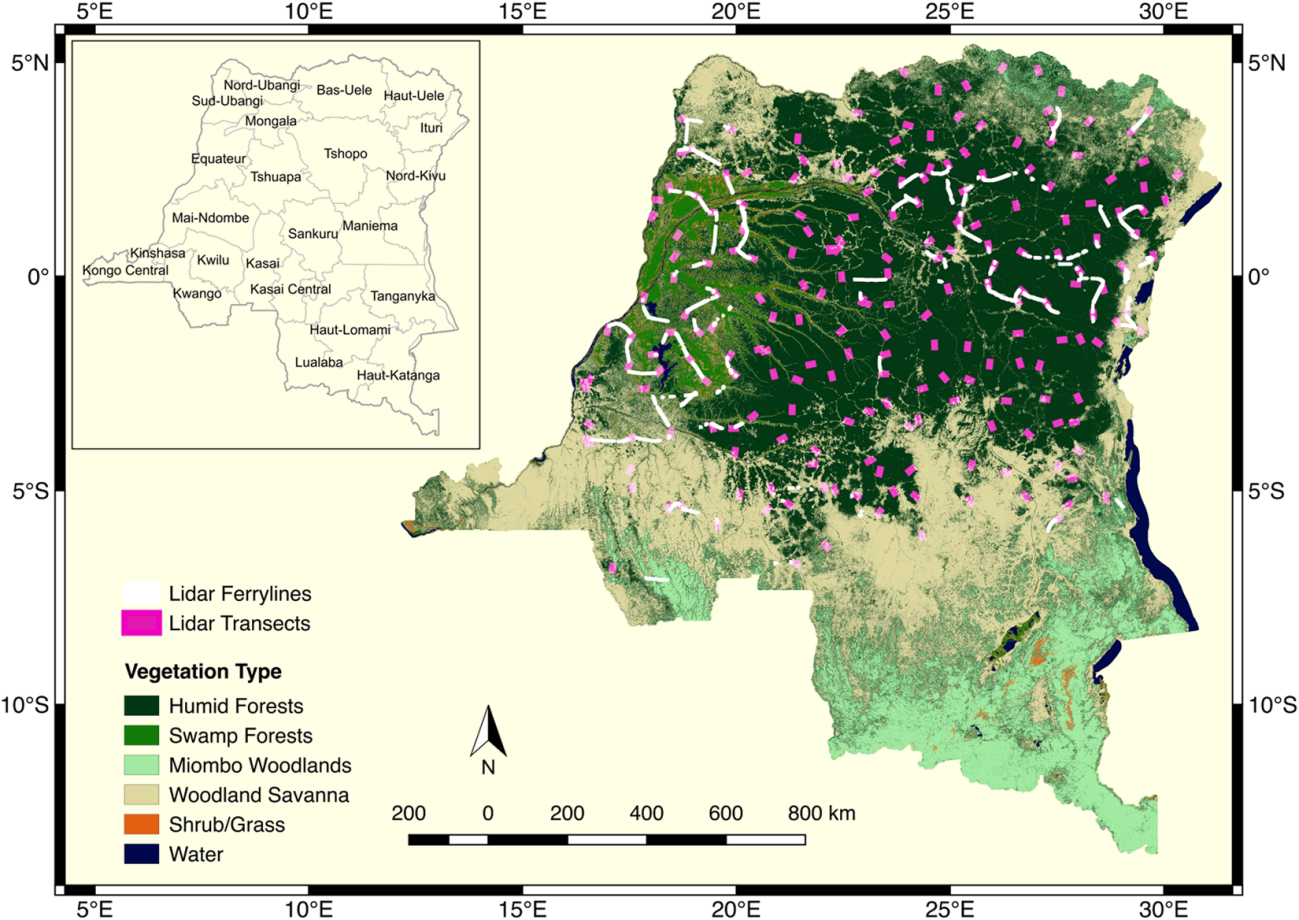
DRC Lidar sampling design over the land cover map. Locations of Lidar transects (~1.5 km × 11 km) and ferry lines between transects are in bold for better display. The map was produced using QGIS v2.8^44^.

LiDAR sampling design followed the methodology introduced under VCS tool VT0005 for using remote sensing observations as inventory techniques for estimating carbon stocks^15^. We created ALS flights, namely the “LiDAR transects”, based on a systematic random sampling design (see Supplementary Methods) where a 1° × 1° grid was overlaid on the forest cover map of the country^21^ produced by Observatoire satellital des forest d’Afrique Centrale (OSFAC). The LiDAR transect locations (Fig. 1) were selected by randomly choosing at least one point within the grid cell. To reduce biomass estimation uncertainty (<1%) at transect level, the transect size was set to approximately 2000 ha based on studies that consider spatial autocorrelations for LiDAR measurements^15,16,22^. The LiDAR transect orientation, and the start and end points also followed randomization of heading angle and location to ensure complete random sampling of the population at each point and to ensure an unbiased inference of the mean and variance of the population (here, AGB). A total of 216 LiDAR transects were flown, providing more than 432,000 ha of forest samples and an additional 150,000 ha samples collected during the ferry flight lines. The sampling units at each of 216 locations are random, suggesting the inference of the forest structure or AGB is independent of the potential spatial correlation between sampling units (LiDAR transects). However, because of the nature of LiDAR sampling from airborne platforms, at each LiDAR transect, the 2000 ha LiDAR data are considered clustered, such that the population mean is the mean of the clustered pixels and the variance must include the spatial correlations existing within each LiDAR transect^15,23–25^.

### LiDAR Biomass Model Estimator

**Figure 2:**
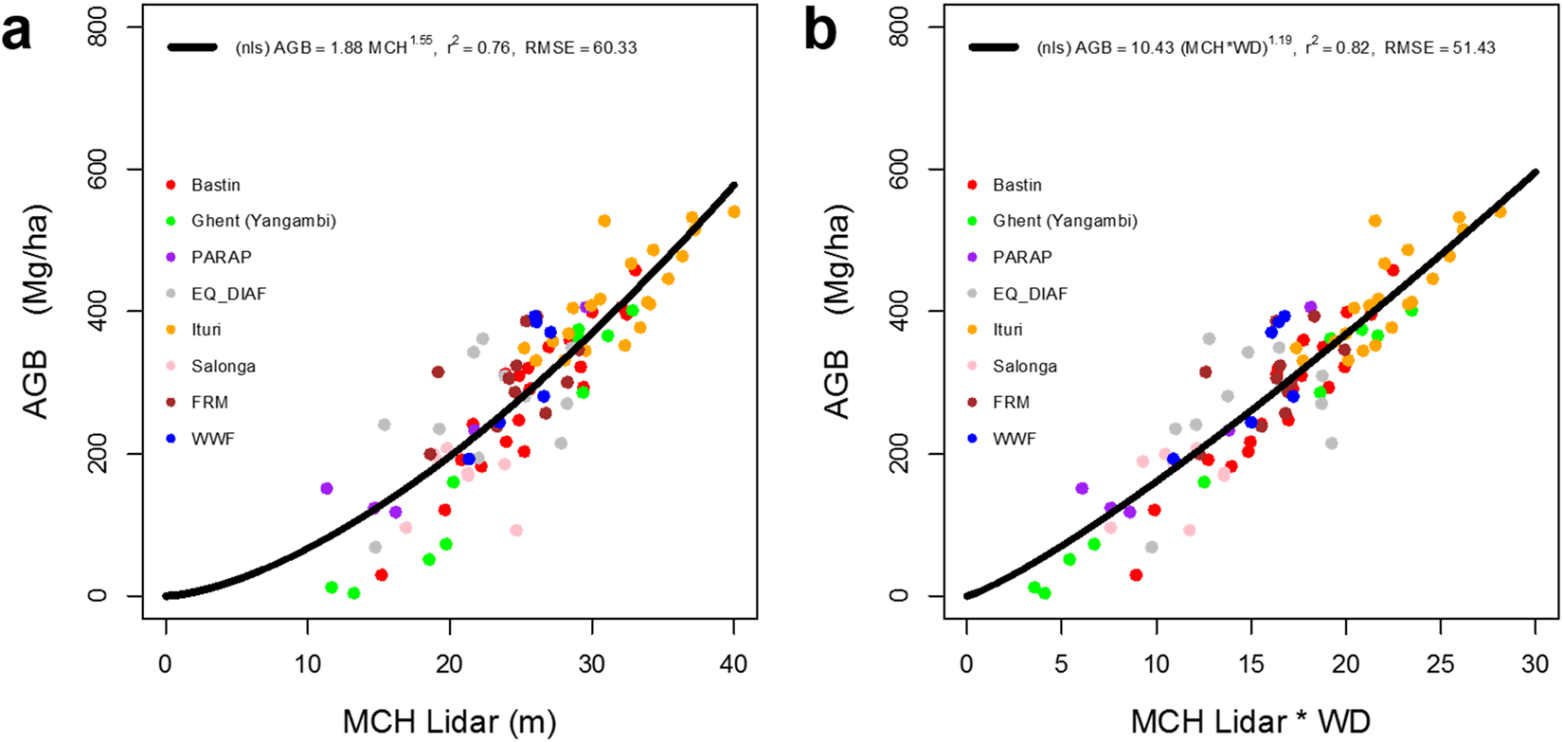
LiDAR-AGB model using 1-ha field plots distributed across DRC. (**a**) AGB model between ground-estimated AGB and Lidar-derived mean canopy height (MCH); (**b**) AGB model between ground-estimated AGB and Wood-density (WD) weighted MCH. The colored points correspond to field sites measured by different research groups (Supplementary Table S1).

The ALS samples were converted to AGB using a non-linear (power-law) model between ground-estimated AGB and LiDAR height metrics across DRC. We used 92 1-ha forest inventory plots (Supplementary Table S1) located in approximately 15% of LiDAR transects to develop the model. The ground plots are mostly located randomly within LiDAR transects and are scattered across DRC (Supplementary Figure S1) to allow developing an unbiased estimator for all forests in the country (see Supplementary Methods). Due to the difficulty of access and cost, the ground sampling was not performed randomly across all LiDAR samples, and therefore, did not completely follow a simple random sampling without replacement. The model (Fig. 2b) is given by:

1 $$AGB=10.43\,{(\overline{WD}h)}^{1.19}$$

where $\overline{WD}$ is the average wood density at each corresponding location based on the forest type at the highest level possible, *h* is the mean top canopy height (MCH) at 1-ha pixels (100 m × 100 m) derived from high resolution (2 m) LiDAR pixels. The scaling constant (10.43), and the power-law exponent (1.19) parameters are derived using a linearized approach to fit the power-law by including a zero-mean Gaussian noise term to account for the uncertainty in measurements. The mean wood density is the only scaling factor that allows the model to be used as an estimator across regions and for all LiDAR pixels (comparing Fig. 2a and b).

### Spatial Prediction of AGB and Carbon Stock

Satellite measurements of surface reflectance sensitive to forest structure and canopy characteristics were used as variables to predict AGB across the landscape using randomly distributed LiDAR-derived AGB samples. We employed the modified Maximum Entropy (MaxEnt) estimator as a non-parametric machine learning algorithm with Bayesian-derived probability functions that allow estimation of mean and variance of AGB at the pixel and regional scales^9,26^ (see Supplementary Methods). MaxEnt produced estimates of AGB at 100 m (1-ha) spatial resolution from Landsat, ALOS PALSAR, and SRTM data. The AGB map and the pixel level uncertainty were used to estimate the mean and variance of aboveground biomass at different forest type and jurisdictional scales. The total carbon in live vegetation was derived by first estimating the belowground biomass (BGB) using allometric models dependent^3,27^ on AGB (Supplementary Information) and using the summation of the two pools and applying the carbon fraction of 0.49 across forest types and regions. The key steps of our spatial prediction include the satellite and LiDAR data processing, spatial modeling using MaxEnt, and uncertainty analysis to provide regional and pixel level estimates of errors associated with the map (Supplementary Fig. S2).

## Results and Discussion

### Forest Biomass Distribution

**Figure 3:**
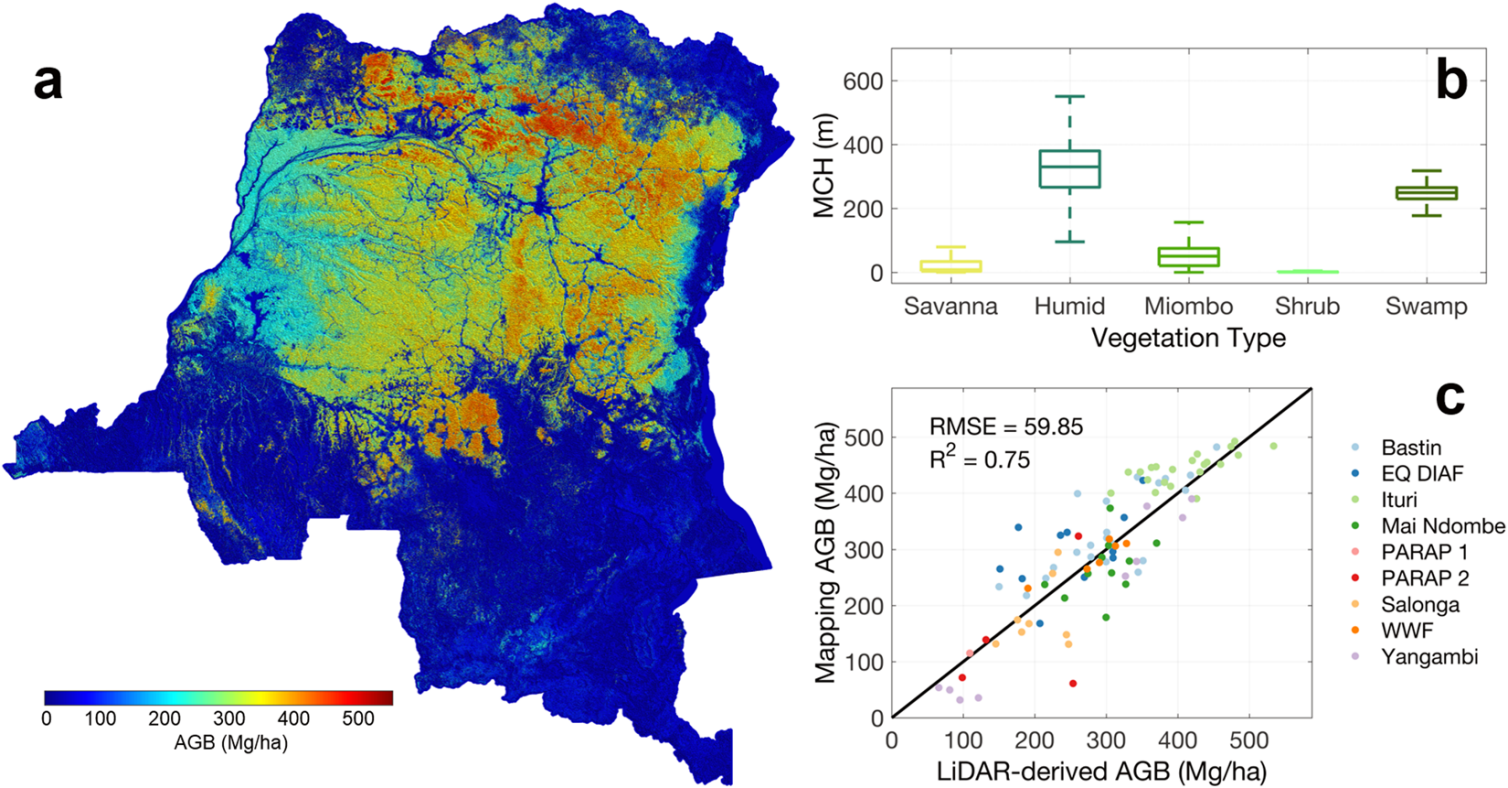
AGB map for DRC and associated analyses. (**a**) Spatial pattern of AGB in the DRC country; (**b**) Mean estimates of AGB for each land cover type; and (**c**) Scatter plot of mapped AGB vs. field LiDAR-derived AGB values. The colormap was generated using MATLAB mapping toolbox^45^.

The AGB map of DRC (Fig. 3) provides the detailed spatial variability of carbon stored in the forests, capturing the physiography of forest structure at landscape and regional scales. At the landscape scale, the variations in AGB follow disturbance and topographical gradients. LiDAR measurements of forest structure capture distribution of large trees and show how degradation, distance to roads and settlements, or geomorphological features associated with slopes, soil type and moisture conditions impact the spatial distribution of aboveground forest structure and biomass^28–31^.

The spatial prediction of AGB provides an unbiased estimate of all available ground plots with a root mean square error (RMSE) of 60 Mg ha^−1^ (Fig. 2c). By averaging AGB across the entire country and applying the carbon fraction of 0.49 of dry matter, we report a mean carbon density of 113 ± 9 MgC ha^−1^ in the aboveground live trees for DRC, consistent with other estimates from bottom-up studies^1^.

The overall variation of AGB across land cover types (see Supplementary Methods) ranges from values as low as 3 (95% Confidence Interval: 0–9) Mg ha^−1^ for savanna and shrublands to as high as 326 (95% CI: 87–476) Mg ha^−1^ for humid forests (Fig. 3b). Within the humid tropical region (Fig. 3a) between the latitudinal bands of 5°S and 5°N, there is spatial variation of carbon storage, showing significant differences by geographical regions.

The largest stretch of high AGB is across the eastern border region of DRC, starting from the northeastern Ituri to nord- and sud-Kivu provinces (Table 1). These forests are distributed over rugged terrains along the foothills of eastern mountains below 1000 m asl (above sea level) and stretch west into Tshopo, Maniema, and southern Sankuru provinces. The average AGB is about 320 Mg ha^−1^ and the AGB values of greater than 450 Mg ha^−1^ are observed over a significant number of 1-ha pixels^32^ (Fig. 2a). Another distinct pattern of high biomass extends in the northern DRC along the remaining intact terra firme forests of Bas-Uele, Nord-Ubangi and Mongala provinces. These forests occupy a relatively flat terrain over Humic acrisols and Hapic Ferralsols soils with mean biomass exceeding 380 Mg ha^−1^ and extensive areas of forests with AGB greater than 500 Mg ha^−1^.

**Table 1:** Biomass and carbon statistics for each province in DRC.

Province	FA1 (Mha)	FA2 (Mha)	AGB Mean (Mg ha^−1^)	Carbon Mean (Mg ha^−1^)	Total AGB (Pg)	Total Carbon (Pg)
Bas-Uele	13.56	12.36	268.93 ± 12.29	162.50 ± 11.11	3.645 ± 0.168	2.203 ± 0.152
Equateur	9.61	9.37	246.09 ± 12.57	148.85 ± 11.05	2.366 ± 0.122	1.431 ± 0.107
Haut-Katanga	6.2	8.22	60.06 ± 8.19	35.48 ± 5.25	0.372 ± 0.052	0.220 ± 0.033
Haut-Lomami	4.22	3.84	80.70 ± 9.89	48.04 ± 6.30	0.340 ± 0.042	0.202 ± 0.027
Haut-Uele	7.45	6.52	173.01 ± 10.70	104.22 ± 8.49	1.289 ± 0.080	0.777 ± 0.064
Ituri	4.68	4.56	312.41 ± 16.00	188.92 ± 13.24	1.461 ± 0.075	0.883 ± 0.062
Kasai	7.18	6.27	249.40 ± 12.17	150.67 ± 10.74	1.792 ± 0.088	1.082 ± 0.078
Kasai Central	3.75	2.86	182.47 ± 11.76	109.95 ± 8.79	0.684 ± 0.045	0.412 ± 0.033
Kasai Oriental	0.12	0.08	83.66 ± 11.71	49.99 ± 7.41	0.010 ± 0.001	0.006 ± 0.001
Kinshasa	0.2	0.08	67.87 ± 10.30	40.45 ± 6.32	0.013 ± 0.002	0.008 ± 0.001
Kongo Central	2.3	0.76	76.49 ± 9.62	45.61 ± 6.10	0.176 ± 0.022	0.105 ± 0.014
Kwango	4.6	3.65	114.68 ± 10.76	68.68 ± 7.25	0.528 ± 0.050	0.316 ± 0.034
Kwilu	3.46	2.59	112.22 ± 9.91	67.39 ± 6.87	0.389 ± 0.035	0.233 ± 0.024
Lomami	1.26	0.86	119.74 ± 10.63	71.85 ± 7.26	0.151 ± 0.014	0.091 ± 0.009
Lualaba	6.93	6.63	89.39 ± 10.04	53.21 ± 6.63	0.619 ± 0.071	0.369 ± 0.047
Mai-Ndombe	10.06	9.42	237.93 ± 12.40	143.85 ± 10.71	2.393 ± 0.126	1.447 ± 0.109
Maniema	10.4	9.75	285.01 ± 12.65	172.29 ± 11.65	2.963 ± 0.133	1.791 ± 0.122
Mongala	5.39	4.97	261.53 ± 13.02	158.10 ± 11.25	1.410 ± 0.071	0.852 ± 0.061
Nord-Kivu	4.71	4.37	253.54 ± 13.59	153.22 ± 11.34	1.194 ± 0.064	0.722 ± 0.054
Nord-Ubangi	3.85	3.37	275.37 ± 14.51	166.45 ± 11.90	1.060 ± 0.056	0.641 ± 0.046
Sankuru	9.31	8.88	310.00 ± 13.83	187.46 ± 12.60	2.885 ± 0.130	1.744 ± 0.118
Sud-Kivu	4.51	4.30	251.25 ± 14.76	151.83 ± 11.60	1.134 ± 0.067	0.685 ± 0.053
Sud-Ubangi	3.85	3.40	212.64 ± 12.83	128.43 ± 10.12	0.819 ± 0.050	0.495 ± 0.039
Tanganyka	6.04	6.77	76.67 ± 8.89	45.60 ± 5.84	0.463 ± 0.054	0.276 ± 0.036
Tshopo	19.82	19.40	323.55 ± 12.99	195.74 ± 12.72	6.413 ± 0.259	3.879 ± 0.254
Tshuapa	13.26	13.11	306.14 ± 13.15	185.21 ± 12.45	4.058 ± 0.176	2.455 ± 0.166
All	166.58	156.26	231.67 ± 9.09	139.90 ± 9.43	38.592 ± 1.529	23.304 ± 1.587

The two FAs (FA1 and FA2; FA stands for “Forested Area”) are values calculated from (1) the LiDAR-derived MCH map, and (2) the land cover map from OSFAC (see Supplementary Methods). The forest pixels for the calculations of AGB and Carbon are determined by the definition of FA1.

The eastern mountains and the northern elevated plateau slope gently towards the interior and to the west of the country where the central depression of the Congo Basin forms the “Cuvette Centrale” swamp forests^33^. The swamp forests cover about 9.5 million hectares in DRC, and are distributed along the Congo and Ubangi rivers, and other large tributaries such as Ruki, Lulonga, Maringa, and Tshuapa river systems, and within the Lake Tumba and Lake Mai Ndombe basins. They have significantly smaller mean carbon density (229, 95% CI: 7–304 Mg ha^−1^) compared to the terra firme humid tropical forests. The swamp forests can also be divided into hardwood and palm dominated swamps, by consulting the classification over an extensive area of peatlands in the Central Congo Basin^34^. The mean AGB for hardwood dominated swamps is 264 ± 21 Mg ha^−1^ and for palm dominated swamp is 71 ± 29 Mg ha^−1^.

On the average, the humid tropical forests of DRC, have much higher biomass density (~300 Mg ha^−1^), higher wood density (~0.66 g cm^3^), and a relatively lower stem density (~400 trees ha^−1^) compared to forests in Amazonia and southeast Asia^7,35,36^. The average AGB in DRC is significantly lower than values reported for African humid tropical forests from research plot networks (~430 Mg ha^−1^) primarily due to differences in region of study and sampling design, but the average wood density and stem density derived from our training plots are approximately the same^35^. The difference in AGB estimates can also be attributed to probabilistic sampling design in DRC (Fig. 1) that unlike research plots in old growth undisturbed forests captures a combination of intact and partially disturbed forests from selective logging.

The results from the analysis of high resolution LiDAR data also suggest that large trees with height exceeding 50 m dominate the areas of high biomass areas. These trees appear to be much larger than average large trees in Amazonia^28,37^. Particularly in monodominant stands captured in the training plot data in Ituri (see Supplementary Methods), there are a number of large trees (e.g. height exceeding 60 m), with mean wood density >0.7 g cm^−3^, AGB values >400 Mg ha^−1^. In general, these majestic forests remain mainly in eastern and northeastern region of DRC with low impact of logging and disturbance, possibly due to the lack of access from political conflicts.

In the southern provinces of DRC, the land cover is dominated by the mosaic of tree grass savanna and riparian forests, extending from the southern Bandudu province towards the extensive southeastern Miombo woodlands in Lualaba and Katanga. These forests have significantly lower AGB (23, 95% CI: 0–113 Mg ha^−1^ and 53, 95% CI: 0–171 Mg ha^−1^, respectively), though they cover an equally large region (117 million ha) in DRC compared to the humid forests (115 million ha). The airborne LiDAR samples were only acquired in the humid tropical forest zone with some coverage in forest-savanna boundary regions in the north and the south of the country, but with almost no data over Miombo woodlands. Similarly, the ground plots used in training the data do not cover the Miombo woodlands. We expect the limited sampling and the higher sensitivity of ALOS PALSAR imagery to woodland biomass range can provide reasonable training data for the machine learning algorithm to estimate spatial distribution of AGB in forests outside the humid tropical zone. In fact, the mean AGB estimates of Miombo woodlands in DRC show very close numbers to ground estimates of other regions in central and southern African countries^13^.

### Uncertainty Estimates

The LiDAR probability sampling approach follows design-based inventory sampling to ensure unbiased estimates of forest structure. However, similar to the national inventory and design-based ground sample plots, the estimation of AGB at local or regional scale depends strongly on the use of an allometric model to convert measurements of structure to biomass. The LiDAR-AGB model plays the same role as the ground allometric model and the overall uncertainty of AGB estimate depends on how well the model was developed. Here, we provide the uncertainty of forest biomass at two levels: (1) We quantify the uncertainty associated with the LiDAR-AGB model using ground plots distributed across DRC; (2) We estimate the uncertainty associated with the MaxEnt prediction at the pixel and jurisdictional scales over the entire country.

#### LiDAR-AGB Model Uncertainty

LiDAR-AGB model was developed using 92 ground plots distributed randomly within LiDAR transects across the country with the condition of feasibility of access or security of the location. We tested for the uncertainty of the model using a bootstrapping (1000 times) cross-validation approach with randomly selecting 80% of data for model fits and 20% for validation. The result suggests that model has a standard error of 52 Mg ha^−1^ but remains relatively unbiased (−0.6 Mg ha^−1^) across all regions. The use of wood density as a weight to LiDAR-derived mean canopy height can help reduce further bias when implementing the model in forests with different tree composition (Fig. 2).

#### Spatial Mapping Uncertainty and Validation

**Figure 4:**
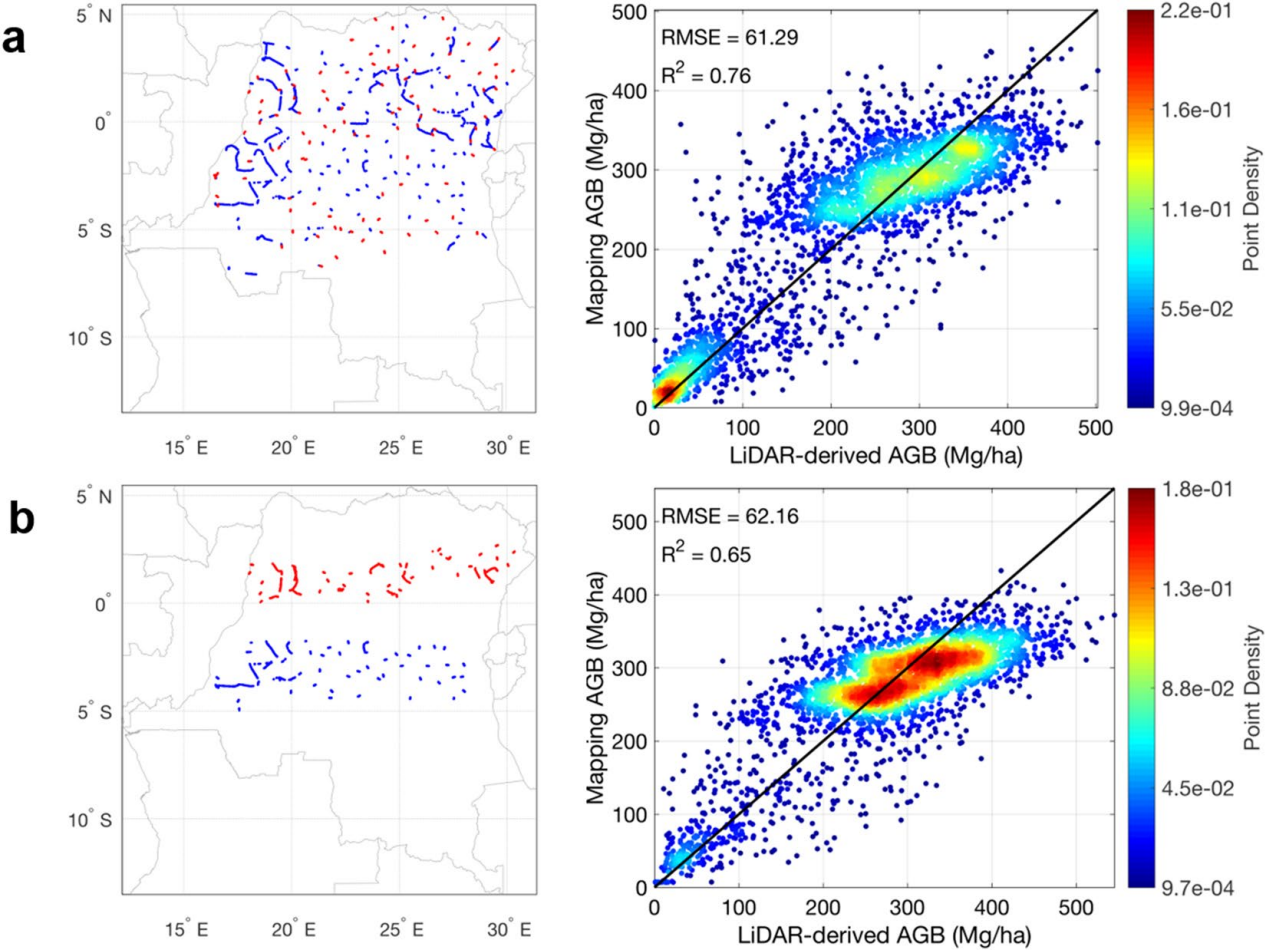
Cross-validation (CV) results from 2 methods. (**a**) CV example of plot-based sampling with the training and test locations on the left panel and the test scatter plot on the right; (**b**) CV example of latitudinal sampling with the training and test locations on the left panel and the test scatter plot on the right. For the left panels, blue dots are the training sample locations and red dots are the test sample locations. The maps were generated using MATLAB mapping toolbox^45^.

We first evaluated the uncertainty associated with the spatial modeling of AGB using cross-validation (CV) approach (see Supplementary Methods). CV results from LiDAR plot-based sampling (Fig. 4a) give the best overall prediction, with an average RMSE of 61 ± 1 Mg ha^−1^ (Supplementary Table S2). Considering the possible existence of residual spatial autocorrelation, CV results from latitudinal sampling (Fig. 4b) have a relatively larger prediction error, with an average RMSE of 70 ± 6 Mg ha^−1^. The two CV results also confirmed that the predictions were statistically unbiased over the entire sample size, with the mean signed deviation (MSD) at 0.4 ± 3.2 Mg ha^−1^ for LiDAR plot-based sampling and −4.5 ± 15.2 Mg ha^−1^ for latitudinal sampling approaches. To further explain the differences between the two CV methods, we used a variogram-based analysis (Supplementary Fig. S3), showing the spatial autocorrelation with paired distance. The spatial autocorrelation in the original AGB map can extend for more than 200 kilometers, and the covariance between spatially close pixels is over 15000. On the other hand, the residuals between our prediction and LiDAR samples show a similar range of spatial autocorrelation but less than 20% of the original covariance. This residual spatial autocorrelation can cause larger prediction uncertainty for pixels far away from the training data, resulting in the differences between two CV methods. This test suggests that we may underestimate the uncertainty in the southern provinces of DRC, where no additional LiDAR and ground samples are currently available in tropical dry and Miombo forests.

Other sources of uncertainty come from (1) the uncertainty of the field-derived LiDAR-AGB model, (2) the geolocation errors between field-derived LiDAR modeling and spatial mapping, and (3) the measurement and interpolation error of airborne LiDAR heights. Here, we estimate the uncertainty relative to the ground-estimated forest biomass from plot level tree inventory and allometric models^38^ (see Supplementary Methods). The LiDAR-AGB model has an average RMSE of 52 Mg ha^−1^ (Fig. 2), which then propagates to the national map with a potential sub-pixel geolocation error. The average sub-pixel geolocation error can be approximated as the nugget effect of zero distance in the semi-variogram analysis (Supplementary Fig. S3), and is roughly 50 Mg ha^−1^. The LiDAR height interpolation error can be modeled using ordinary kriging (Supplementary Fig. S4). Under the original 2-meter resolution for LiDAR raster product (see Supplementary Methods), we found that regions without adequate ground returns could have uncertainty as high as 1 meter in forest height. However, the spatial aggregation of 2-meter products to 1-ha resolution makes this part of uncertainty rather small and negligible. Therefore, compared to aforementioned sources of uncertainty, LiDAR height measurements provide the most accurate estimation assuming that ground points can truly represent the ground.

**Figure 5:**
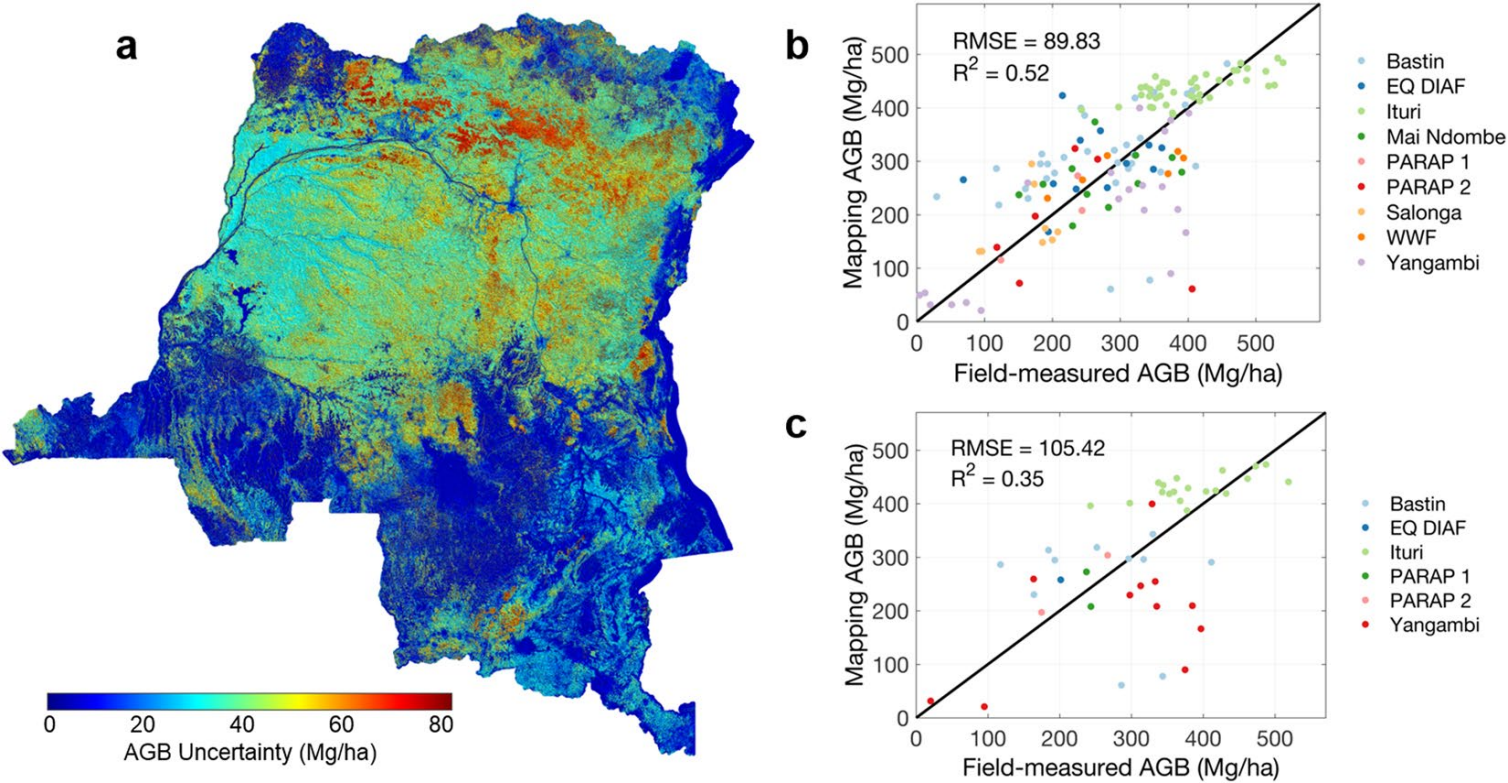
AGB validation scatter plots. (**a**) AGB uncertainty map with one standard deviation. (**b**) Validation against all available field measurements; (**c**) Validation against all independent field sites. The names and locations of the field sites can be found in Supplementary Table S1. The colormap was generated using MATLAB mapping toolbox^45^.

The spatial modeling uncertainty of AGB represented by pixel level prediction error is the last source of uncertainty (Fig. 5a). The results show that majority of the AGB modeling uncertainty of tropical forests is bounded between 40 to 90 Mg ha^−1^. However, compared to pixel values with ground-estimated AGB at the 1-ha plots, the uncertainty is larger (~90 Mg ha^−1^) when compared to all field plots (Fig. 5b), and about 105 Mg ha^−1^ when compared with an independent data set (Fig. 5c). If we assume different processes impacting the uncertainty of our AGB map are unrelated, the propagation of uncertainty from field-derived LiDAR AGB modeling error (~50 Mg ha^−1^), pixel mismatch error (~50 Mg ha^−1^), to the average spatial mapping error (~70 Mg ha^−1^), is theoretically about 100 Mg ha^−1^, similar to what we found from independent field validation (Fig. 5c).

### National Carbon Statistics

We report the carbon and biomass estimates for each province in DRC, to provide baselines for future forest management or emission reduction projects (Table 1). Results show that 4 provinces (Tshuapa, Tshopo, Ituri and Sankuru) have the highest mean AGB of more than 300 Mg ha^−1^. The 10 other provinces (Mai-Ndombe, Equateur, Sud-Ubangi, Nord-Ubangi, Mongala, Bas-Uele, Nord-Kivu, Sud-Kivu, Maniema, and Kasai) have mean AGB estimates around 200 Mg ha^−1^. These 14 provinces possess 75% of the total carbon in the country. The remaining 12 provinces have lower AGB density and total carbon (AGB + BGB) than the others, and nevertheless, contain more than 30% of the country’s forested area. The provinces with the lowest AGB density are Kinshasa, Kasai Oriental, Lomami and Kongo Central, which contribute less than 1% to the country-level carbon storage. At national level, we have the mean AGB of 232 ± 9.1 Mg ha^−1^ for all forested regions, with about 4% average modeling error when considering errors of regional estimates using model-based inference^14^ (see Supplementary Methods).

The total carbon estimate for each province is different from mean values due to the area-weighted nature. We report Tshopo, Tshuapa and Bas-Uele to be the top 3 provinces of carbon storage, each containing more than 2 PgC due to its large area of tropical forests. Mai-Ndombe, Equateur, Maniema and Sankuru also have 1.4–1.8 PgC in each province with a large forest coverage of over 9 to 10 Mha. However, the forest type is also important to estimating total carbon. Note that Lualaba, Tanganyka and Haut-Katanga all have 6–7 Mha of forests, comparable to the forest coverage in Haut-Uele and Kasai, but the total carbon storage is only 0.3 PgC – approximately one third of the total carbon in those provinces. The total carbon of the entire DRC is around 23.3 ± 1.6 PgC. The modeling error for this total number is ~7%, larger than the mean AGB estimates due to the uncertainty associated with the belowground biomass calculations. The uncertainty of total carbon also varies with both the mean uncertainty and the number of pixels. For example, Bas-Uele and Mongala have similar estimates of mean AGB and the associated errors, but the forested region in Bas-Uele is more than double of Mongala, causing the total carbon uncertainty in Bas-Uele much larger than Mongala.

### Environmental Controls

The climate and edaphic characteristics in DRC may partly explain the spatial variability of forest carbon stocks. By upscaling our carbon density map to a quarter-degree, matching the spatial resolution of available products for climate and soil variables, we found weak but significant relationships between carbon stocks and environmental variables.

**Figure 6:**
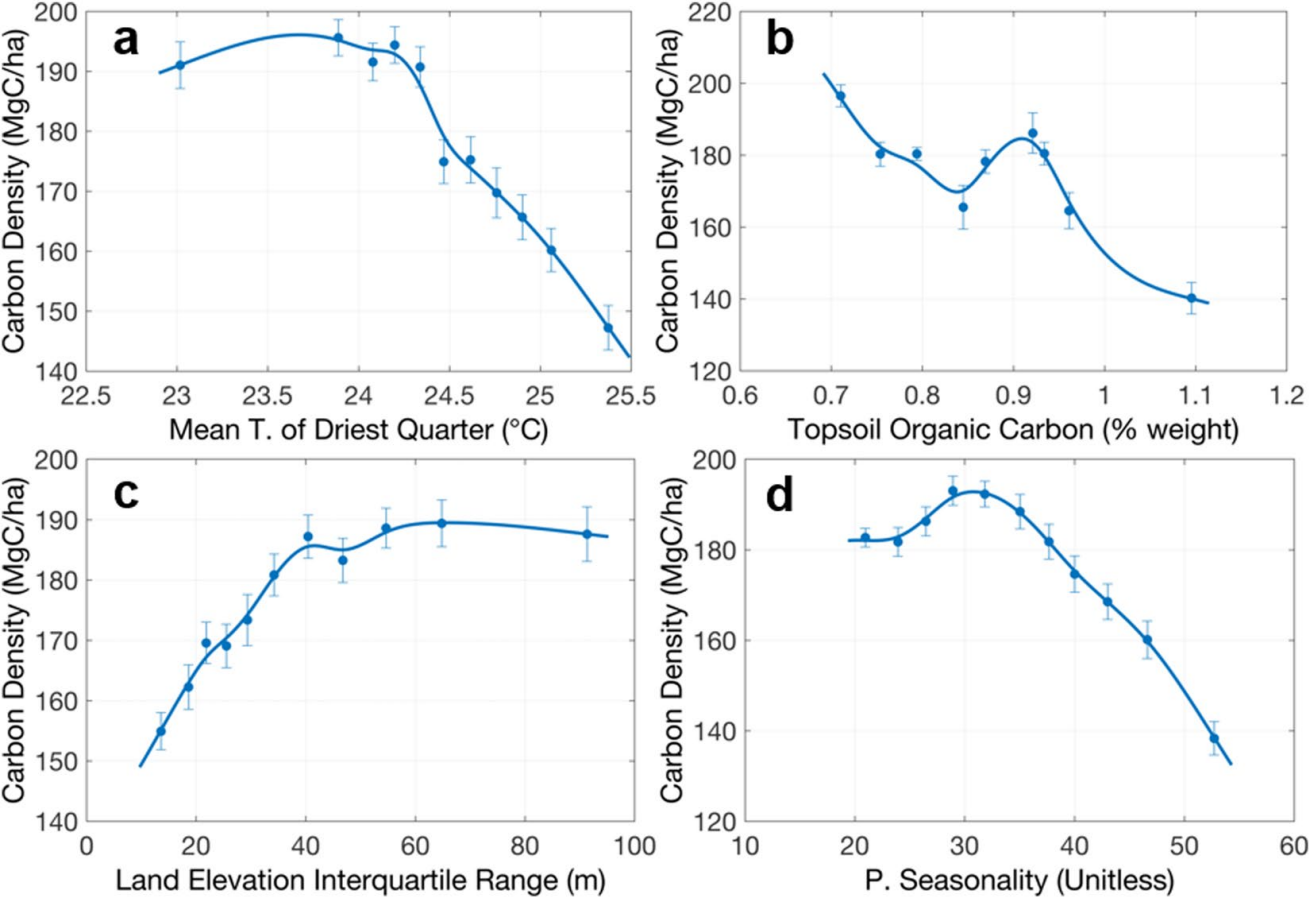
Selected mean relationships between carbon density and environmental variables in humid tropical forests in DRC. Panels show carbon density vs. (**a**) mean temperature of driest quarter, (**b**) topsoil organic carbon, (**c**) land elevation variation, and (**d**) precipitation seasonality. The plots show the relationships between mean values within each interval of environmental variables. The errorbar associated with carbon density is the standard error of mean estimation from bootstrapping samples. See Supplementary Methods for detailed data description.

For humid forests in DRC, our analysis shows the most important environmental variables for determining spatial distribution of carbon are mean temperature of driest quarter, topsoil organic carbon, land elevation variation and rainfall seasonality. These 4 variables explain about 28% of the carbon stock variation. Although the power of explanation is not very strong, likely due to the heterogeneity of forest structure, composition, and other soil-related factors missing in this study, all 4 variables significantly regulate the distribution of carbon at least from the mean characteristics (Fig. 6). The mean temperature of the driest quarter is the most important variable (Fig. 6a), showing negative correlation with carbon density and suggesting that areas with higher temperature, associated with lowland forests and with larger disturbance, have less carbon storage than forests at lower temperature associated with higher elevation and less disturbed forests. This may also follow the observations that forests have an optimum range of temperature for CO_2_ uptake^39,40^. Rainfall seasonality (Fig. 6d) is also well correlated with carbon density showing a similar negative relationship to indicate that more carbon is stored in less seasonal forests. Interestingly, among soil properties, the topsoil organic carbon also plays an important role for carbon storage of Congo basin (Fig. 6b), consistent with our findings in the tropical forests of the Amazon Basin^31^, which shows a significant negative effect of soil organic carbon to dominant tree height. One possible explanation is that soil carbon, controlled by pH values, is strongly affected by forest species composition^41^. The peatlands found in Congolese swamp forests^34^ can also explain this relationship, by showing increasing topsoil carbon, associated with water-logged or peatlands have smaller trees. Higher temperatures in tropical forests near swamp-dominated areas could also protect soil organic carbon from decomposition^42^. The variation in land elevation, related to various landscape structure, affecting incoming solar radiation, hydrological features, as well as soil compounds, shows a positive relationship with carbon density (Fig. 6c), meaning more forest carbon stored over complex terrains. This effect may also be due to the higher probability of forest degradation and logging in areas with flat terrain that was out of the scope of this study and could not be verified.

**Figure 7:**
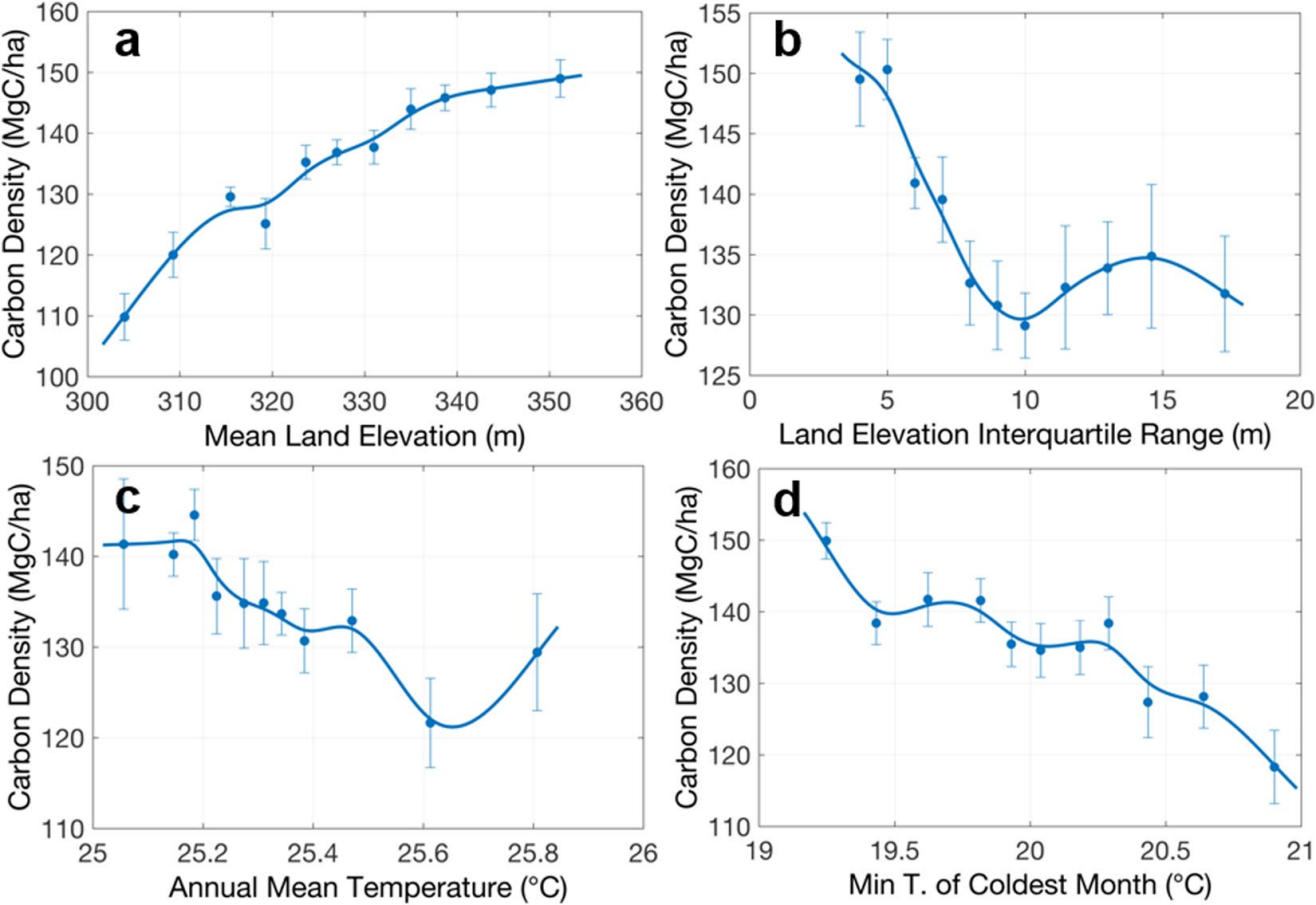
Selected mean relationships between carbon density and environmental variables in Swamp forests of DRC similar to Fig. 5. Panels show carbon density vs. (**a**) mean land elevation, (**b**) land elevation variation, (**c**) annual mean temperature, and (**d**) minimum temperature of coldest month.

The swamp forests, mainly distributed along the Congo river system, have a more predictable pattern related to environmental variables. Results show that 66% of the carbon spatial variation in these forests can be explained by 4 variables: mean land elevation, land elevation variation, annual mean temperature and minimum temperature of coldest month (Fig. 7). The most important variable, mean land elevation, can explain about 49% of the carbon variation (Fig. 7a), showing a linear increase of carbon stock density with elevation over the range of about 50 m. Unlike terra firme humid forests, swamp forests show a negative relationship between carbon and elevation variation (Fig. 7b), suggesting a higher density of swamp forests growing on the flat terrain that allows permanent or seasonal inundation over the year. The annual mean temperature (Fig. 7c) in swamp forests is correlated with minimum temperature of the coldest month (Fig. 7d), and temperature diurnal range and annual range. The negative correlation with carbon density is consistent with what we found in the humid forests. The soil properties in these forests are highly correlated with each other and temperature variables, probably due to their similar geographical distribution, and therefore, could not significantly explain the variations of carbon density in swamp forests.

**Figure 8:**
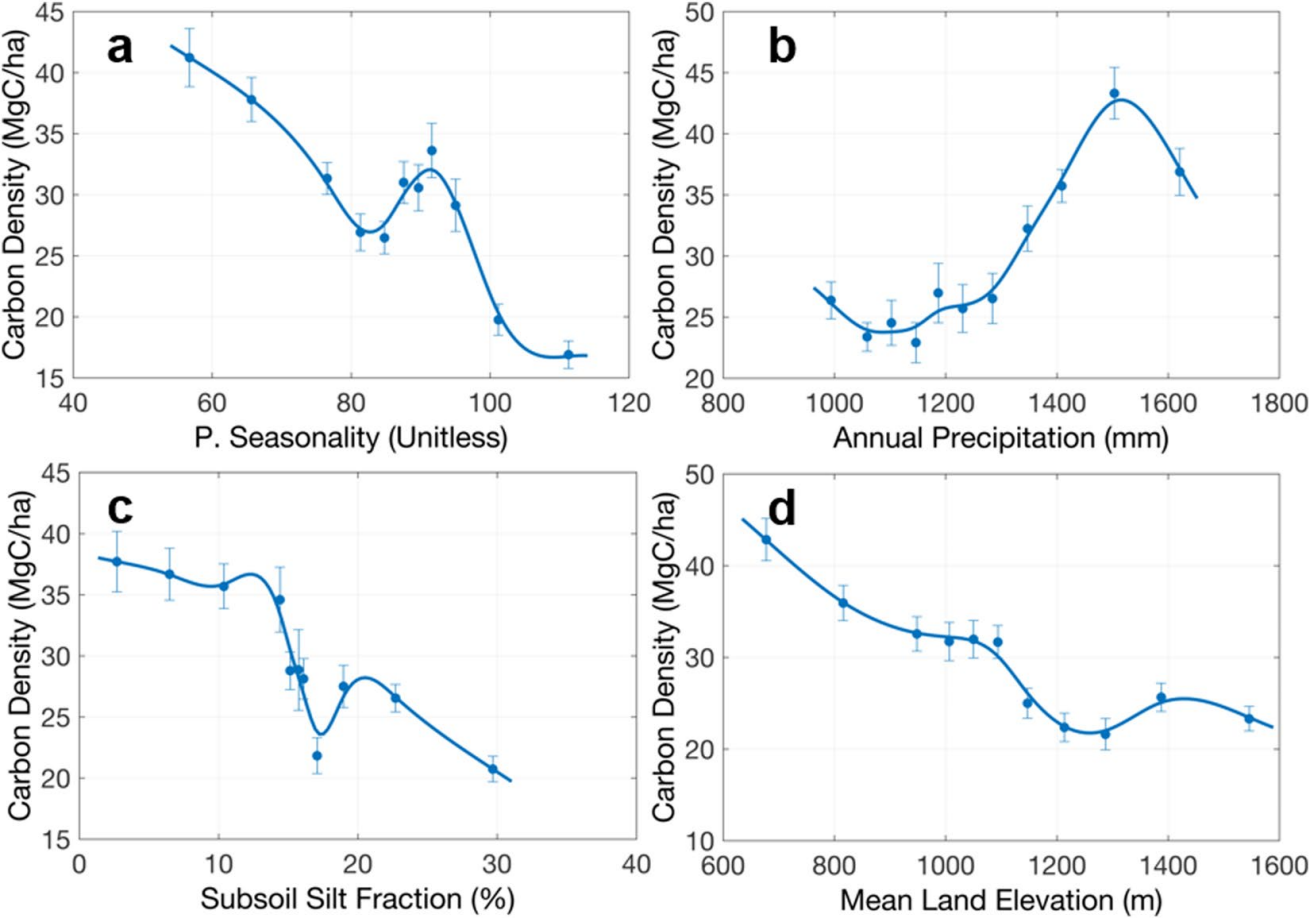
Selected mean relationships between carbon density and environmental variables in Miombo woodlands in DRC similar to Fig. 5. Panels show carbon density vs. (**a**) precipitation seasonality, (**b**) annual precipitation, (**c**) subsoil silt fraction, and (**d**) mean land elevation.

The Miombo woodlands in DRC cover mostly the southern part of the country. Compared to humid and swamp forests, these forests have much lower carbon density values. As expected, there are distinct environmental variables that determine the variations of forest carbon in these forests (Fig. 8). Rainfall seasonality becomes the most important variable (Fig. 8a), followed by annual precipitation (Fig. 8b), subsoil silt fraction (Fig. 8c), and mean land elevation (Fig. 8d). No temperature variable plays an important role in the carbon distribution. But since these seasonal forests are distributed over a large range of elevation, we found that most temperature seasonality features are correlated strongly with precipitation seasonality, and mean temperature features are tightly correlated with mean land elevation. The collinearity of soil characteristics suggests that the topsoil silt fraction and similarly the sand faction in the top soil can equally explain the distribution of Miombo forests. The soil silt fraction is also correlated with the soil nutrient availability such as the cation-exchange capacity, and the soil organic carbon, together positively impacting forest biomass accumulation.

**Figure 9:**
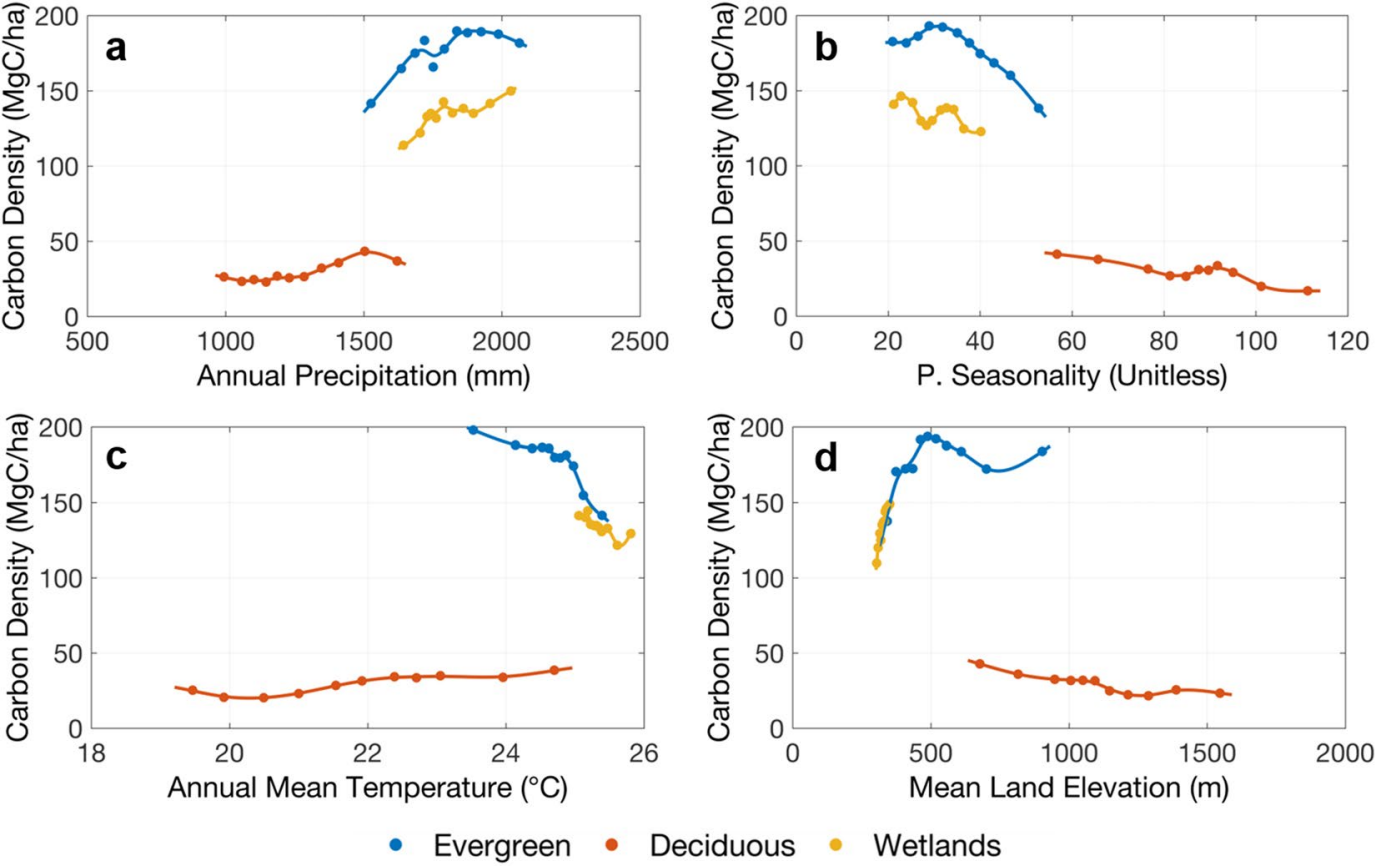
Mean relationships between carbon density and environmental variables for three main forest types in DRC. Panels show carbon density vs. (**a**) annual precipitation, (**b**) precipitation seasonality, (**c**) annual mean temperature, and (**d**) mean land elevation. The plots show the relationships between mean values within each interval of environmental variables.

Plotting features which are generally important to forest carbon distribution (Fig. 9), we found that both annual precipitation (Fig. 9a) and precipitation seasonality (Fig. 9b) contribute in separating tropical humid/swamp forests and sub-tropical Miombo woodlands, consistent with the cross-species study in global tree heights^43^. The annual precipitation separates the tropical/sub-tropical forests at a threshold around 1500 mm, and the separation of the 2 types is ever clearer in the precipitation seasonality figure at around 55. However, rainfall itself cannot differentiate the humid and swamp forests. Using annual mean temperature (Fig. 9c) and mean land elevation (Fig. 9d), swamp forests are at the tail of the curve for humid forests in each figure. This suggests that swamp forests exist in regions with low elevation and high temperature as expected, with precipitation patterns similar to those in terra firme humid forests.

## Conclusion

The systematic and probability based inventory of forest structure with airborne LiDAR data provided the first physiographical variations of the forest height and carbon density at landscape scales in the Congo Basin. Using the LiDAR inventory measurements trained with ground plots, we were able to develop the national-level forest biomass distribution along with uncertainty in the second largest country after Brazil with tropical forests. The sampling density was designed to provide sub-national and province-level carbon statistics, as well as AGB estimates summarized by forest types. By examining the climate and edaphic variables, we identified key climate (temperature and precipitation), terrain (elevation and interquartile range) variables, and soil properties contributing to spatial distribution of forest carbon stocks and forest types. The development of carbon estimates and the national map follows a verifiable methodology with formal uncertainty quantification that can be applied to other tropical countries for cost-effective and efficient assessment of forest carbon storage and changes at regional and national levels.

## Electronic supplementary material

Supplementary Information
