# Supplementary material for: Spatial Distribution of Carbon Stored in Forests of the Democratic Republic of Congo
Source: Sci Rep. 2017 Nov 8;7:15030. doi: 10.1038/s41598-017-15050-z (PMC5678085; doi:10.1038/s41598-017-15050-z)
Supplement: Supplementary file 1 — Supplementary Information [file 41598_2017_15050_MOESM1_ESM.pdf]

SUPPLEMENTARY INFORMATION

Spatial Distribution of Carbon Stored in Forests of the Democratic Republic of Congo

Liang Xu[1], Sassan S. Saatchi[1,2], Aurélie Shapiro[3], Victoria Meyer[2], Antonio Ferraz[2], Yan Yang[1], Jean-Francois Bastin[4,5], Norman Banks[6], Pascal Boeckx[7], Hans Verbeeck[8], Simon L. Lewis[9,10], Elvis Tshibasu Muanza[11], Eddy Bongwele[12], Francois Kayembe[13], Daudet Mbenza[13], Laurent Kalau[13], Franck Mukendi[13], Francis Ilunga[13], Daniel Ebuta[13]

[1]*Institute of Environment and Sustainability, University of California, Los Angeles, CA, USA.*

[2]*Jet Propulsion Laboratory, California Institute of Technology, Pasadena, CA, USA*

[3]*World Wide Fund for Nature(WWF) Germany Biodiversity Unit, Berlin, Germany*

[4]*Landscape Ecology and Plant Production Systems Unit, Université libre de Bruxelles, Belgium*

[5]*BIOSE department, Gembloux Agro Bio Tech, Gembloux, Belgium*

[6]*Southern Mapping Company, Airborne LiDAR Survey Unit, Johannesburg, South Africa*

[7]*Isotope Bioscience Laboratory - ISOFYS, Ghent University, Ghent, Belgium*

[8]*CAVElab – Computational and Applied Vegetation Ecology, Ghent University, Ghent, Belgium*

[9]*School of Geography, University of Leeds, Leeds, UK*

[10]*Department of Geography, University College London, London, UK*

[11]*World Wide Fund for Nature (WWF) Kinshasa, DR Congo*

[12]*Observatoire Satellital des Forets d'Afrique Central (OSFAC), Kinshasa, DR Congo*

[13]*Direction des Inventaires et Aménagement Forestiers (DIAF), Kinshasa, DR Congo*

Supplementary Methods

AGB Model from Inventory Plots

Ground inventory plots distributed throughout DRC were collected to calibrate the airborne LiDAR data (Supplementary Fig. S1). All plot data were collected between 2011 and 2016. The requirement for the field data included two sets of plots: (1) permanent plots at 1-ha (100 m x 100 m) and (2) four auxiliary plots at 0.25 ha (50 m x 50 m) distributed on the eastern and western axis from the center of the permanent plots at 250 m and 500 m intervals. However, the ground plots collected by partners varied in size, shape and design because they were collected from different sources and collaborators. We received a total of 4684 ground plots, varying between sizes of 0.04ha and 1ha. 139 of these plots meet our 1ha plot size requirements for biomass model calibration, of which 47 are set aside for independent validation (ground estimated AGB vs Maximum Entropy estimated AGB) because they did not fall into any LiDAR scene. The smallest plots could not be used for calibration of the LiDAR data because of their size and potential geolocation uncertainty. However, smaller plots were nevertheless used to retrieve information on wood density, assuming that plot size does not affect this metric.

All locations of the plots where data was collected were selected randomly in the LiDAR transects to make sure that the process of the validation does not introduce any artificial bias. We also used pre-existing plots such as plots from Dr. Bastin and Smithsonian, where some plots fall within the LiDAR scenes. In all cases, the measurement of the trees followed the standard protocols provided by the IPCC guidelines: Within each plot, all stems > 10 cm in diameter (D) at breast height (dbh) were measured at 130 cm from ground or above the butresses or trunk deformations.

In addition, the total heights (H) of all trees were measured using instruments such as the LaserACE 2D Hypsometer (MDL, York, UK) (i.e. Bastin plots). At YANGAMBI plots, all tree individuals were classified into diameter classes (that is, 10–20, 20–30, 30–50, >50 cm) and all species contributing to 95% of the basal area of the plot were selected for further measurements. For each of these species, two individuals were selected within each diameter class for tree height measurements using a Nikon Laser Rangefinder Forestry Pro hypsometer[1]. Height of trees that were not measured in the field was estimated using local DBH-H models, typically for each study site. DBH-H allometric models have the form:

$$H = a \times \ln(D) + b \quad (1)$$

However, some sites did not have any measured tree heights and had no neighboring plots that could be used to assign tree height. For trees in these sites (Ituri, PARAP Luhudi and PARAP Maniema), AGB was estimated using a different allometric equation (see below, Eq. 2).

Tree species were identified and recorded for wood density calculations. Wood density was assigned using the Global Wood Density Database (GWDD) for tropical trees[2,3] and the FAO database, at the highest level possible, species level being the most precise level, followed by genus and family level. In the cases where tree identification was missing or did not match any name in our databases, the mean wood density of the plot was assigned. At YANGAMBI plots, wood samples of all important species were taken for the calculations of wood density.

Plot location is known for all the plots and is characterized by either the center of the plot (circular plots, with known radius) or the four corners of the plots (square or rectangular plots). Although the accuracy of geolocation is supposed to be between 3 and 8m, additional error may

accrue when calibrating the LiDAR data with ground data, especially when small plots are used. Also, larger plots include a larger number of trees, reducing the overall uncertainty of biomass estimation[4]. Finally, it has been shown that using ground plots <1ha decreases the accuracy of LiDAR models because of edge effects[5]. Therefore, plots of 1ha only were used to calibrate our LiDAR model.

Specific allometric equations for Central African forests are rare because of the lack of available destructive sampling data of trees of different size without rigorous statistical approaches for developing the regression models and testing for random errors and bias[6–8]. One of the most reliable allometric equations widely used in most studies in tropical forests is developed by Chave et al. (2005)[9], improving upon earlier equation by Sandra Brown (1997)[10] . More recently Chave et al. (2014)[6] improved the pan-tropical allometric model using a total of 4004 harvested trees with trunk diameter ranging from 5 to 212 cm distributed over a wide range of vegetation types from tropical to sub-tropical, woodland savanna and both undisturbed and secondary forests. The new equation includes a large number of trees from Central Africa, improved over Chave et al. (2005)[9], which only included trees from Asia and Americas. The mean percent bias and variance of this model was only slightly higher than that of locally fitted models[6]. Wood specific gravity was an important predictor of aboveground biomass, especially when including a much broader range of vegetation types than previous studies.

Here, AGB was calculated using the allometric models developed by Chave et al, (2014)[6].

$$AGBH_{est} = \frac{10^{-3}}{A}\sum_{i=1}^{N} 0.0673 \times (\rho_i D_i^2 H_i)^{0.976} \quad (2)$$

$$AGB_{est} = \frac{10^{-3}}{A}\sum_{i=1}^{N} \exp(-1.803 - 0.976\,E + 0.976\ln(\rho_i) + 2.673\ln(D_i) - 0.0299(\ln(D_i))^2)$$

Where $AGBH_{est}$ and AGB_{est} are the estimated above ground biomass in units of Mg ha^{-1}, A is the area of the plot in hectare (ha), D_i is the diameter of each tree in the plot in centimeter (cm), H_i is the height of each tree in meter (m), and ρ_i is the wood density of each tree in g cm^{-3}. E is a measure of environmental stress, taking into account temperature seasonality, precipitation seasonality and climatic water deficit, at any location on the globe[6]. AGB_{est} was used for sites where no tree height measurement or estimation was available (Ituri, PARAP Luhudi and PARAP Maniema).

The aboveground biomass was further augmented for all trees with DBH < 10 cm. Trees < 10 cm in diameter and height > 1.3 m were measured in Ituri plots and few plots outside of DRC in Gabon and Republic of Congo. We developed an equation from 1-ha plots in DRC and Gabon where trees with DBH > 1cm have been measured in the field. Small trees will add approximately 3-7% on the average to the aboveground biomass values. The equation below converts the AGB estimates for trees > 10 cm ($AGB_{>10cm}$) to AGB estimate for all trees with DBH > 1 cm ($AGB_{>1cm}$). The number of 1-ha plots used in the model are 40, and the allometry between biomass of small trees and large trees had a tight relationship with $R^2 > 0.99$, suggesting a relatively negligible error in scaling up the biomass of forests in DRC to capture the biomass of small trees.

$$AGB_{>1cm} = 1.872 \times AGB_{>10cm}^{0.906} \quad (3)$$

LiDAR Biomass Estimator

Calibration plots of 1ha were used to develop the LiDAR-biomass model. Out of the 139 plots of 1ha available in DRC, 92 plots of 1ha fall into a LiDAR image. Each of these plots represents a region of interest (ROI) in our LiDAR 2m resolution CHM dataset. For each ROI, pixels were extracted and used to calculate the LiDAR mean canopy height (MCH) of each plot.

Calibration of LiDAR data consists in finding the relationship between LiDAR MCH and ground estimated AGB across DRC (See Supplementary Fig. S1, Supplementary Table S1). We tested a model that includes wood density as a weighting parameter to allow the model to be used for different forest types with different average wood density. The overall form of the model is:

$$AGB = a(MCH \times WD)^b + \varepsilon \quad (4)$$

where WD represents the plot mean value of wood density in units of g cm^{-3}, MCH is in units of m from LiDAR observation, and $\varepsilon \sim N(0, \sigma^2)$ represents the uncertainty in measurements.

Belowground Biomass Estimation

We encountered virtually no consistent measurements of belowground biomass in our data compilation efforts. This result was not surprising, as it is not practical to measure below ground biomass in most tropical forests on a routine basis. It is also very difficult to develop an appropriate, country-specific allometric equation for root biomass. Methods for collecting

belowground biomass data are laborious, time-consuming, and technically challenging to perform correctly. Instead, belowground biomass is usually estimated from aboveground biomass using regression equations developed from field data collected across multiple biomes. A synthesis of data from available literature, along with elimination of data collected using unclear or incorrect methods, provided a universal equation for estimating forest belowground biomass[11]. The equations below show how the belowground biomass (BGB) can be estimated from AGB for humid tropical forests:

$$BGB = 0.235 \times AGB,\ \ if\ AGB > 125\ Mg\ ha^{-1}$$
$$BGB = 0.205 \times AGB,\ \ if\ AGB\ \leq 125\ Mg\ ha^{-1} \tag{5}$$

For all forest types, we use:

$$BGB = 0.489 \times AGB^{0.89} \tag{6}$$

National Carbon Mapping

To produce the national carbon map in 1-ha (100-meter) spatial resolution, we built a synergistic model for estimating biomass and total carbon from a variety of data sources, including the *in-situ* measurements of key forest attributes such as AGB and wood density, airborne small-footprint LiDAR sampling with a wider spatial coverage at the country level, as well as the spatial data from contemporary satellite imagery covering the DRC country in the optical and microwave domain. Supplementary Fig. S2 demonstrates the basics and key steps of our workflow.

Nation-level Mapping Data Preprocessing

Nation-level Input feature layers in our study include satellite data and ancillary mapping products of administrative boundary and major land cover types in the country of DRC. We used the Congo basin forest atlas data from the Ministère de l'environnement et Développement Durable (MEDD) to define the country boundary[12]. From the source vector data in shapefile format, we rasterized the map in 100-meter resolution with binary numbers. We assigned the value of 1 to each pixel within the country boundary, and the value of 0 to the rest pixels. The final output is under the geographic coordinate system (GCS) with WGS84 datum (pixel resolution in 0.0008983°). This becomes our reference 1-ha map, and every other data set was registered to this map.

We possess continuous coverage of medium-resolution satellite data products over the study region. We used three sources of satellite data as our inputs to the synergistic model. The first input is the mosaic of Landsat-8 top-of-atmosphere (TOA) reflectance data averaged from April 2013 to August 2016. We used the simple cloud-score algorithm on the Google Earth Engine[31] for cloud screening, and kept the median values over the 3 years as valid observations. The final cloud-free imagery of Landsat-8 contains 4 bands including band 4 (Red), 5 (NIR), 6 (SWIR-1), and 7 (SWIR-2) at 30-meter spatial resolution. To account for the BRDF (bidirectional reflectance distribution function) effect of Landsat data, we corrected our Landsat-8 mosaic using the MODerate-resolution Imaging Spectroradiometer (MODIS) Nadir BRDF-Adjusted Reflectance (NBAR) product (MCD43A4)[14,15]. By obtaining the MODIS NBAR mosaic similar to Landsat-8 for the same time period, we applied a simple correction as follows:

$$L_{corr} = L \times \frac{N_{fmean}}{L_{fmean}} \tag{7}$$

where L and L_{corr} are the Landsat reflectance before and after the correction, L_{fmean} and N_{fmean} are the focal means of original Landsat and NBAR reflectance, and we used the window size of 2500x2500 meters for both data sets. The second input is the digital elevation model (DEM) data derived from the Shuttle Radar Topography Mission (SRTM). The global mosaic of SRTM land elevation product[16] has a spatial resolution of 30 meters processed by the National Aeronautics and Space Administration (NASA). Although the latest release of SRTM land elevation is the void-filled product (SRTM v3), there are regions with missing data in DRC (e.g. regions in the eastern part of the country (Supplementary Fig. S5a). We used the ASTER GDEM v2 (Global Digital Elevation Model Version 2) data[17] to further fill the gaps (Supplementary Fig. S5b) in these areas. The third input is the radar backscatter data from the Phased Array L-band Synthetic Aperture Radar (PALSAR) sensor aboard the Advanced Land Observing Satellite "DAICHI" (ALOS). ALOS has the L-band SAR observations at the wavelength of 1270 MHz for five years' operation from January 2006 to May 2011. The Japan Aerospace Exploration Agency (JAXA) has produced the ortho- and slope-corrected backscattering coefficient of PALSAR global mosaics in both HH and HV polarizations from 2007 to 2010[18]. We used the 4-year mean (2007-2010) of PALSAR as our last set of satellite input data.

The preprocessing of satellite data includes spatial aggregation and image registration. We aggregated 4 bands of Landsat-8 mosaic, 2 bands (HH/HV) of ALOS PALSAR, and the SRTM v3 DEM data into 100-meter spatial resolution using spatial average. We also kept the local standard deviation of SRTM data as an additional layer, creating a final set of satellite inputs with 8 layers. Using the 1-ha reference map created from the MEDD country boundary, we registered all our satellite layers to the same raster grid.

To have a prior knowledge on the vegetation types of DRC, we used the land cover (LC) product from the Climate Change Initiative (CCI) project of the European Space Agency (ESA)[19]. The CCI-LC map has a global coverage in 300-meter spatial resolution and has been validated using ground and high resolution imagery. The new release (version 1.6.1) has improved the representation of cropland patterns in the Democratic Republic of Congo[19]. In this study, we used the map of the latest epoch 2010 (from 2008 to 2012) and developed a higher resolution map at 1-ha to match the other data layers used in estimating forest carbon density. The resampling approach was done by selecting a set of high-confidence pixels (more than 80% confidence level provided by the quality map) as training data for generating a new 1-ha stratification map over DRC. From the original 36 number of land cover types, we found 6 dominant classes in the country (Fig. 1). Using randomly sampled LC pixels with high confidence, we built the Random Forest classification[20] model from 8 satellite layers, and generated the 6-class land stratification map in 100-meter resolution. We used this map as an additional categorical layer to the input of spatial modeling. The stratified map was compared with the original map at 300-m resolution and no discrepancy in spatial patterns and land use types was detected. This approach preserved the land cover patterns of the original map and provided a 100-m map without the artifacts that are introduced from direct resampling from 300 m to 100 m resolution. We also used the dedicated national-level land cover map produced by Observatoire satellital des forest d'Afrique Centrale (OSFAC) as an additional source of validation.

LiDAR data preprocessing

We received airborne small-footprint LiDAR measurements from DRC with a total of 216 plots and 96 Ferry lines (Fig. 1). It covers the entire tropical forest region of DRC with spatially

balanced samples. The Southern Mapping Company (SMC) conducted the airborne LiDAR survey and scanned the ground using the Optech ALTM 3100 LiDAR scanner from June 2014 to February 2015. The resulting point cloud of LiDAR data has an average density of 4 pts per m^2. The preprocessing of LiDAR data from SMC includes trajectory calculation, LiDAR point calibration, and LiDAR point classification (separating ground and vegetation points). The final classified LiDAR points were delivered in LAS format to the University of California, Los Angeles (UCLA).

We created three raster products in 2-meter spatial resolution from the LiDAR point cloud. (1) We used the existing classification labels from SMC, and created the digital terrain model (DTM) using mean elevation of LiDAR points labeled as class 2 (ground) in each 2-meter pixel. Pixels with missing data were interpolated by natural neighbor interpolation. (2) LiDAR points classified as 2 (ground), 3 (low vegetation), and 4 (medium vegetation) were used all together to create digital surface model (DSM). The maximum elevation of the used LiDAR points in each 2-meter pixel was picked as the DSM value. (3) As a result, the canopy height model (CHM) was calculated as the height difference between DSM and DTM.

The decision of creating 2-meter raster products was based on the designed airborne data acquisition in DRC. With an average coverage of approximately 4 LiDAR points per square meter, raster creations in 1-meter resolution are not appropriate. We found unexpected stripes of data gaps from 1-meter CHM (Supplementary Fig. S6a), because of missing vegetation LiDAR points in these pixels. These data gaps cannot be corrected by the spatial interpolation method, because ground points exist in some of these gaps (Supplementary Fig. S6b). Such ground points in gaps lead to extremely low CHM values compared to nearby pixels, while they are apparently canopy pixels observed from aerial photos (Supplementary Fig. S6c). We decided to use the 2-

meter spatial resolution when a sufficient number of LiDAR points are available in each pixel to determine the maximum height. Results show that the CHM raster in 2-meter resolution can eliminate most of the undesired gap signals without sacrificing too much spatial detail (Supplementary Fig. S6d).

The 2-meter CHM products were further used to create the 1-ha map of LiDAR-measured mean canopy height (MCH). For 1-ha (100-meter) spatial resolution, we have approximately 2500 2-meter observations for each pixel. To ensure that the MCH values can well represent the mean characteristics of 1-ha pixels, we set MCH observations valid only when 90% of the 1-ha pixels are covered by airborne LiDAR measurements, i.e., we excluded most of the edge pixels with partial coverage in the 1-ha mapping process. The final LiDAR-derived 1-ha map of DRC has a total of ~665K valid pixels that can be used in national-level MCH and carbon mappings.

Spatial Modeling

With the availability of nation-level feature layers and LiDAR-derived MCH, we were able to build a supervised learning model as the spatial estimator to predict the unknown MCH for locations where we have environmental data. Maximum Entropy (ME), as a supervised learning algorithm, is a probability-based algorithm that seeks the probability distribution by maximizing the information contained in the existing measurements[21,22]. In the ME algorithm, a measurement A of class k has the probability of occurrence $p(A_k)$ with the constraint that probabilities of all $p(A_k)$ must sum to 1 ($\sum_k p(A_k) = 1$). From information theory, the most uncertain probability distribution is the one that maximizes the entropy term:

$$E = -\sum_{k} p(A_k) \ln p(A_k) \quad (8)$$

With knowledge of additional information, i.e. the training set of MCH measurements with corresponding input feature data X, the probability distributions are "conditioned" on the available observations:

$$p(A_k|X) = p_k(X)p_0(A_k)/p(X) \quad (9)$$

The right part of the above equation follows the Bayes' theorem, meaning that the posterior probability $p(A_k|X)$ depends on the distribution of X and equals to the product of prior probability $p_0(A_k)$ and the probability distribution $p_k(X)$ that finds X to be in the class k, and normalized by the probability distribution of X for the entire domain of measurement variables (nation-level mapping layers). For our metric MCH, we categorize the numeric values into a set of classes: $k_1, k_2, k_3, \ldots, k_n$, where $0 < k_1 \leq MCH_1 < k_2 \leq MCH_2 < \cdots < k_n \leq MCH_{max}$. And each class has a nominal value of MCH – usually the mean value of each class, $\overline{MCH}_k$. To predict the MCH value for any pixel i with known measurements X_i, we calculate it as the expectation of all classes given the ME results retrieved from the training set:

$$\langle MCH_i \rangle = \frac{\sum_{k=1}^{N} p(A_k|X_i)\overline{MCH}_k}{\sum_{k=1}^{N} p(A_k|X_i)} \quad (10)$$

Empirical tests have found that the model performs better when assigning higher weights to more probable classes,

$$\langle \mathrm{MCH_i} \rangle = \frac{\sum_{\mathrm{k=1}}^{\mathrm{N}} [p(A_k|X_i)]^{\mathrm{m}} \overline{\mathrm{MCH}}_{\mathrm{k}}}{\sum_{\mathrm{k=1}}^{\mathrm{N}} [p(A_k|X_i)]^{\mathrm{m}}} \tag{11}$$

The parameter optimization procedure suggests m = 3 as the best parameter with the smallest average relative error and keeping most test points aligned with the 1-to-1 line[23,24]. We evaluated 3 statistical measures in our parameter tuning procedure, including the coefficient of determination (R^2), the root-mean-square error (RMSE), and the mean signed deviation (MSD). Besides the overall MSD applied to all test samples, we assessed two additional MSD measures for both small trees (MSD1) and large trees (MSD2). We define MSD1 as the MSD calculated for test samples with the sum of predicted MCH and measured MCH to be less than 20 meters. Similarly, MSD2 is defined as MSD for samples with the sum of predicted MCH and measured MCH to be larger than 60 meters. Results also suggest that we use a relatively larger background number to avoid overfitting.

The mapping of WD adopted the same spatial modeling procedure as MCH. But WD training data were from field measurements of 1-ha plots covering the whole country (Supplementary Fig. S1). A total of 4287 1-ha WD samples were used as ground truth, and extrapolated using the same bias-corrected RF estimator to the country-level map.

The LiDAR-derived allometric model of AGB (Eq. 4) is expressed as a power-law function with MCH and WD as input variables. With the availability of both MCH and WD maps at the country level, we can produce the final AGB map by applying the allometric equation.

Uncertainty Assessment

Cross Validation

Cross validation (CV) is a modeling technique used to check the statistical learning consistency using the independent data from the training set itself[25,26]. It can not only be used to check the performance of the spatial modeling by making predictions on new data that are never used in the training, but also often used as a technique of parameter tuning[27] to avoid "overfitting". For regression-based analysis, we normally use the mean-squared-error (MSE) as the scoring parameter in the CV process. There are several ways of cross validation commonly used to evaluate the performance, including k-fold approach, leave-one-out CV, repeated random subsampling (or Monte Carlo CV), and so on[28,29].

Considering the existence of spatial autocorrelation, we used 2 types of Monte Carlo CV approaches: (1) *Plot-based sampling*. To avoid the test samples being dependent on the training within the same LiDAR transect (short-distance spatial autocorrelation), our subsampling procedure was based on plot numbers, i.e., once LiDAR transects were used as test samples, we would not use any LiDAR measurement within these plots as training. (2) *Latitudinal sampling*. To avoid the test samples in one plot being dependent on the training samples from nearby plots (long-distance spatial autocorrelation), we sampled the training and test sets based on latitudinal bands, so that the test samples were the farthest from training samples. This approach finds the lower bound of our spatial modeling, and guarantees that most pixels at the country level should have predictions within the uncertainty range. In every repetition of these 2 CV test, we randomly sampled 5000 observations as training and 3000 observations as test samples.

Spatial Autocorrelation

We used the variogram plot to demonstrate the existence of spatial autocorrelations[25,30]. The variogram-based approaches assume that the spatial autocorrelation of variables only depends on

the distance (h), while it has no other directional or locational dependence. The Variogram ($\gamma(h)$) is defined as

$$\gamma(\mathrm{h}) = \frac{1}{2}\mathrm{E}\left[\left(\mathrm{y}_{\mathrm{x_i}} - \mathrm{y}_{\mathrm{x_j}}\right)^2\right] = [C(0) - C(h)], \quad \text{where}\left\|\left(\mathrm{x_i} - \mathrm{x_j}\right)\right\| = h \tag{12}$$

where $C(h)$ is the covariagram depending on the distance h. The investigation of variogram and covariagram of model residuals can check the model performance on removing the spatial dependence of the original data.

Pixel Uncertainty and Regional Uncertainty

Our spatial estimator (ME) inherently provides pixel-level mapping uncertainty as the sum of absolute errors between the predicted value ($\hat{y}$) and the nominal value of each class ($\bar{y}_k$) weighted by retrieved probabilities $p(A_k)$,

$$\sigma_\varepsilon = \frac{\sum_k |\hat{y} - \bar{y}_k| p(A_k)}{\sum_k p(A_k)} \tag{13}$$

To assess the uncertainty of regional mean, we followed Chen et al. 2016 for regional estimates[31],

$$\bar{\sigma}^2 = \frac{1}{N^2}\left(\sum_{i=1}^{N}\sum_{j=1}^{N}\mathrm{cov}\left(\sigma_{\varepsilon,i}\,,\sigma_{\varepsilon,j}\right)\right) + \frac{1}{N^2}\left(\sum_{i=1}^{N}\sum_{j=1}^{N}\mathrm{cov}\left(\sigma_{f,i}\,,\sigma_{f,j}\right)\right) + \frac{1}{N^2}\left(\sum_{i=1}^{N}\sum_{j=1}^{N}\mathrm{cov}\left(\sigma_{z,i}\,,\sigma_{z,j}\right)\right) \tag{14}$$

where N is the total number of pixels, σ_ε, σ_f and σ_z are the pixel-level errors from 1) spatial mapping uncertainty, 2) allometric equation uncertainty, and 3) uncertainty of predictor variables (often LiDAR height retrievals), respectively. The three sources of errors are assumed independent, so that the overall uncertainty of regional estimates comes from the three covariance terms.

We model the first covariance using spatial autocorrelation,

$$\bar{\sigma}_\varepsilon^2 = \frac{1}{N^2}\left(\sum_{i=1}^{N}\sigma_i^2 + \sum_{i=1}^{N}\sum_{j(j\neq i)}^{N}\rho_{ij}\sigma_i\sigma_j\right) \tag{15}$$

where ρ_{ij} is the correlation coefficient between pixels i and j, and it can be approximated from the variogram (Eq. 12) normalized $C(h)$ under the assumption that spatial autocorrelation only changes with distance h.

The second covariance is related only to the allometric model coefficients and can be reformulated as,

$$\bar{\sigma}_f^2 = \sum_{p=1}^{m}\sum_{q=1}^{m}\left(\frac{1}{N}\sum_{i=1}^{N}\frac{\partial f}{\partial \phi_p}\right)\mathrm{cov}(\phi_p\,,\phi_q)\left(\frac{1}{N}\sum_{j=1}^{N}\frac{\partial f}{\partial \phi_q}\right) = \sum_{p=1}^{m}\sum_{q=1}^{m}\left(\bar{g}_p\mathrm{cov}(\phi_p\,,\phi_q)\bar{g}_q\right) \quad (16)$$

where $\bar{g}_p = \frac{1}{N}\sum_{i=1}^{N}\frac{\partial f}{\partial \phi_p}$ is the mean of first derivative with respect to the allometric model coefficient ϕ_p, and m is the total number of coefficients in the allometric model – in our LiDAR-AGB model (Eq. 4), m equals to 2.

The third covariance is related to the LiDAR-derived height measurement errors – in our LiDAR-AGB model, σ_z is the WD-weighted LiDAR mean canopy height. Without in-situ validation of height measurements, it is impossible to evaluate this type of error. Discussion in the main paper has shown that at least the model-based height interpolation is very accurate and the error in 1-ha resolution is negligible. Chen et al 2016 also assumes the LiDAR metric errors are statistically independent[31]. We thus conclude that the third covariance contributes very little in the regional uncertainty estimates (often thousands or millions of 1-ha pixels), and has been set to zeros in the regional uncertainty calculations.

Environmental Controls

Climate and Soil Data

To evaluate the environmental control of the DRC tropical forests, we used several data sets produced from different research groups. The climate data were obtained from the WorldClim climate database[32,33] for our study. WorldClim is a set of average monthly climate data collected globally from ground-based weather stations and interpolated to a 1-km resolution grid. Variables 1 to 11 are related to characteristics of temperature, including annual mean/min/max

temperatures and seasonality parameters such as diurnal and annual range, standard deviation and seasonal mean temperatures. Variables 12 to 19 capture the characteristics of precipitation similar to the variables used for temperature. The average bioclimatic variables are derived from different sources spanning over three decades of observations (1960 to 1990).

The soil data were based on the Harmonized World Soil Database (HWSD) and were used to evaluate the impact of edaphic conditions on forest height distribution. The HWSD data are gridded in a 30 arc-second (about 1km) raster format with over 16000 soil-mapping units collected from the Food and Agriculture Organization of the United Nations (FAO), the International Institute for Applied Systems Analysis (IIASA), and other partners[34]. The soil maps are linked to an attribute database of 12 top- or sub- soil characteristics, including physical compositions such as clay and sand contents, chemical properties such as pH values and cation-exchange capacity, as well as biological variables such as organic matters. Four original source databases, the European Soil Database (ESDB), the China soil map (CHINA), the regional Soil and Terrain (SOTER) databases (SOTWIS) and the Digitized Soil Map of the World (DSMW), were used to create the HWSD raster files through harmonization and merging processes including range and missing data checks, recoding, unit conversions, data inconsistency checks, and so on[35].

For terrain topography, we used the surface elevation data from the Shuttle Radar Topography Mission (SRTM) collected on a near-global scale using Interferometric Synthetic Aperture radar (InSAR) measurement at C-band (5.3 GHz)[16,36]. The original data of SRTM has a spatial resolution of ~30 meters (1 arcsec).

The high-resolution original data were resampled from approximately 90-meter (3 arcsec) to 1-km (30 arcsec) spatial resolution using spatial average as well as the local variation (interquartile range), both of which were used as environmental layers representing terrain characteristics.

Data Processing and Regression Model Selection

To compare different environmental variables with our retrieved forest carbon density values, we resampled the original high-resolution data to 0.25°x0.25° spatial resolution, using the spatial average for all climate, soil and elevation variables, the local standard deviation for an extra layer of land elevation, and the majority resampling for the land-cover map. Based on the aggregated land cover, we tested the impacts of environmental variables on retrieved carbon density in the tropical evergreen forests, wetland forests, and the sub-tropical deciduous forests. After removing the low-quality data and outliers, we obtained a total of 1059 valid quarter-degree samples in evergreen forests, 111 samples in wetland forests, and 319 samples in deciduous forests. For our interested response variable, forest carbon density, we got 33 environmental inputs, including 19 WorldClim variables, 12 soil attributes, and 2 land elevation features (Supplementary Table S3). For the current study, we simply wanted to identify the important environmental variables which are linearly related to forest carbon density, without considering multi-collinearity, spatial autocorrelation, and non-linear effects. To achieve this goal, we used the Lasso (least absolute shrinkage and selection operator) regression[26] which performs both variable selection and regularization to find the best minimum set of predictor features. Through the 10-fold cross-validation procedure using all available training data, we identified the best 6 environmental variables that give the best prediction accuracy for forest carbon density of each selected land cover type (Supplementary Table S4). For the top-4 selected important variables, we also checked the multicollinearity of predictor variables using variance

inflation factor (VIF). A VIF exceeding 10 is the sign of serious multicollinearity requiring correction. Our analyses do not show signs of multicollinearity for the final selected variables.

Supplementary References.

1. Kearsley, E. *et al.* Conventional tree height–diameter relationships significantly overestimate aboveground carbon stocks in the Central Congo Basin. *Nat. Commun.* **4,** 2269 (2013).
2. Chave, J. *et al.* Towards a worldwide wood economics spectrum. *Ecol. Lett.* **12,** 351–366 (2009).
3. Zanne, A. *et al.* Data from: Towards a worldwide wood economics spectrum. *Ecol. Lett.* (2009). doi:doi:10.5061/dryad.234
4. DeWalt, S. J. & Chave, J. Structure and Biomass of Four Lowland Neotropical Forests. *Biotropica* **36,** 7–19 (2004).
5. Meyer, V. *et al.* Detecting tropical forest biomass dynamics from repeated airborne lidar measurements. *Biogeosciences* **10,** 5421–5438 (2013).
6. Chave, J. *et al.* Improved allometric models to estimate the aboveground biomass of tropical trees. *Glob. Change Biol.* **20,** 3177–3190 (2014).
7. Djomo, A. N., Ibrahima, A., Saborowski, J. & Gravenhorst, G. Allometric equations for biomass estimations in Cameroon and pan moist tropical equations including biomass data from Africa. *For. Ecol. Manag.* **260,** 1873–1885 (2010).
8. Ngomanda, A., Lebamba, J., Engone-Obiang, N. L., Lepengue, N. & M'Batchi, B. Caractérisation de la Biomasse sèche des mosaïques forêt-savane des plateaux Okouma et Bagombé au sud-est du Gabon. *J. Appl. Biosci.* **68,** 5147–5428 (2013).
9. Chave, J. *et al.* Tree allometry and improved estimation of carbon stocks and balance in tropical forests. *Oecologia* **145,** 87–99 (2005).
10. Brown, S. Estimating biomass and biomass change of tropical forests: a primer. *FAO For. Pap. FAO* (1997).

11. Mokany, K., Raison, R. J. & Prokushkin, A. S. Critical analysis of root : shoot ratios in terrestrial biomes. *Glob. Change Biol.* **12,** 84–96 (2006).
12. WRI. Congo Basin Forest Atlases. *Democratic Republic of Congo | World Resources Institute* (2013). Available at: http://www.wri.org/our-work/project/congo-basin-forests/democratic-republic-congo#project-tabs. (Accessed: 26th August 2016)
13. Google Earth Engine. Landsat Algorithms | Google Earth Engine API. *Google Developers* (2016). Available at: https://developers.google.com/earth-engine/landsat. (Accessed: 4th October 2016)
14. Schaaf, C. B. *et al.* First operational BRDF, albedo nadir reflectance products from MODIS. *Remote Sens. Environ.* **83,** 135–148 (2002).
15. Schaaf, C. B., Liu, J., Gao, F. & Strahler, A. H. Aqua and Terra MODIS Albedo and Reflectance Anisotropy Products. in *Land Remote Sensing and Global Environmental Change* (eds. Ramachandran, B., Justice, C. O. & Abrams, M. J.) 549–561 (Springer New York, 2011).
16. Farr, T. G. *et al.* The Shuttle Radar Topography Mission. *Rev. Geophys.* **45,** RG2004 (2007).
17. NASA JPL. ASTER Global Digital Elevation Model [Data set]. (2009).
18. Shimada, M. *et al.* New global forest/non-forest maps from ALOS PALSAR data (2007–2010). *Remote Sens. Environ.* **155,** 13–31 (2014).
19. ESA CCI. New release of 300 m global land cover and 150 m water products (v.1.6.1) and new version of the User Tool (3.10) for download. *ESA CCI Land cover website* (2016). Available at: http://www.esa-landcover-cci.org/?q=node/169. (Accessed: 26th August 2016)
20. Breiman, L. Random Forests. *Mach. Learn.* **45,** 5–32 (2001).

21. Berger, A. L., Pietra, V. J. D. & Pietra, S. A. D. A Maximum Entropy Approach to Natural Language Processing. *Comput Linguist* **22,** 39–71 (1996).

22. Phillips, S. J., Anderson, R. P. & Schapire, R. E. Maximum entropy modeling of species geographic distributions. *Ecol. Model.* **190,** 231–259 (2006).

23. Saatchi, S. S. *et al.* Benchmark map of forest carbon stocks in tropical regions across three continents. *Proc. Natl. Acad. Sci.* **108,** 9899 (2011).

24. Yu, Y. Global Distribution of Carbon Stock in Live Woody Vegetation. (University of California, Los Angeles, 2013).

25. Kanevski, M., Pozdnoukhov, A. & Timonin, V. *Machine Learning for Spatial Environmental Data: Theory, Applications, and Software*. (EPFL Press, 2009).

26. Hastie, T., Tibshirani, R. & Friedman, J. *The Elements of Statistical Learning: Data Mining, Inference, and Prediction*. (Springer Science & Business Media, 2013).

27. Segal, M. R. Machine Learning Benchmarks and Random Forest Regression. *Cent. Bioinforma. Mol. Biostat.* (2004).

28. Kohavi, R. A Study of Cross-validation and Bootstrap for Accuracy Estimation and Model Selection. in *Proceedings of the 14th International Joint Conference on Artificial Intelligence - Volume 2* 1137–1143 (Morgan Kaufmann Publishers Inc., 1995).

29. Refaeilzadeh, P., Tang, L. & Liu, H. Cross-Validation. in *Encyclopedia of Database Systems* (eds. LIU, L. & ÖZSU, M. T.) 532–538 (Springer US, 2009). doi:10.1007/978-0-387-39940-9_565

30. Isaaks, E. H. & Srivastava, R. M. *An Introduction to Applied Geostatistics*. (Oxford University Press, 1990).

31. Chen, Q., McRoberts, R. E., Wang, C. & Radtke, P. J. Forest aboveground biomass mapping and estimation across multiple spatial scales using model-based inference. *Remote Sens. Environ.* **184,** 350–360 (2016).

32. Hijmans, R. J., Cameron, S. E., Parra, J. L., Jones, P. G. & Jarvis, A. Very high resolution interpolated climate surfaces for global land areas. *Int. J. Climatol.* **25,** 1965–1978 (2005).

33. Synes, N. W. & Osborne, P. E. Choice of predictor variables as a source of uncertainty in continental-scale species distribution modelling under climate change. *Glob. Ecol. Biogeogr.* **20,** 904–914 (2011).

34. Nachtergaele, F. O. F. & Licona-Manzur, C. The Land Degradation Assessment in Drylands (LADA) Project: Reflections on Indicators for Land Degradation Assessment. in *The Future of Drylands* (eds. Lee, C. & Schaaf, T.) 327–348 (Springer Netherlands, 2008). doi:10.1007/978-1-4020-6970-3_33

35. FAO/IIASA/ISRIC/ISSCAS/JRC. Harmonized World Soil Database (version 1.2). (2012).

36. Rabus, B., Eineder, M., Roth, A. & Bamler, R. The shuttle radar topography mission—a new class of digital elevation models acquired by spaceborne radar. *ISPRS J. Photogramm. Remote Sens.* **57,** 241–262 (2003).

37. QGIS Development Team. *QGIS Geographic Information System*. (Open Source Geospatial Foundation, 2017).

38. NASA JPL. NASA Shuttle Radar Topography Mission Global 1 arc second [Data set]. (2013).

39. GDAL Development Team. *GDAL - Geospatial Data Abstraction Library, Version 2.2.0*. (Open Source Geospatial Foundation, 2017).

Supplementary Figures

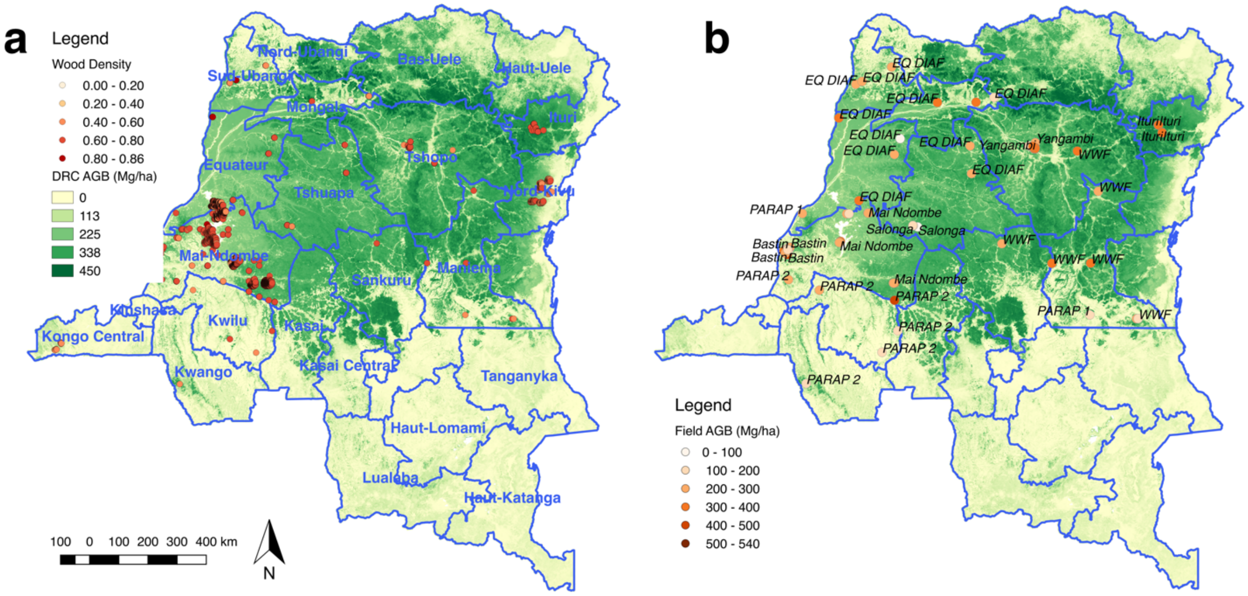

Supplementary Figure S1. Locations of available field data in DRC. (a) All available wood density field data in DRC. (b) All available 1ha field plots of AGB measurements. The colored base map is our prediction map of AGB, the blue labels on the left panel are province names, and the black labels on the right panel are the source names of field measurements. Maps were produced using QGIS v2.8[37].

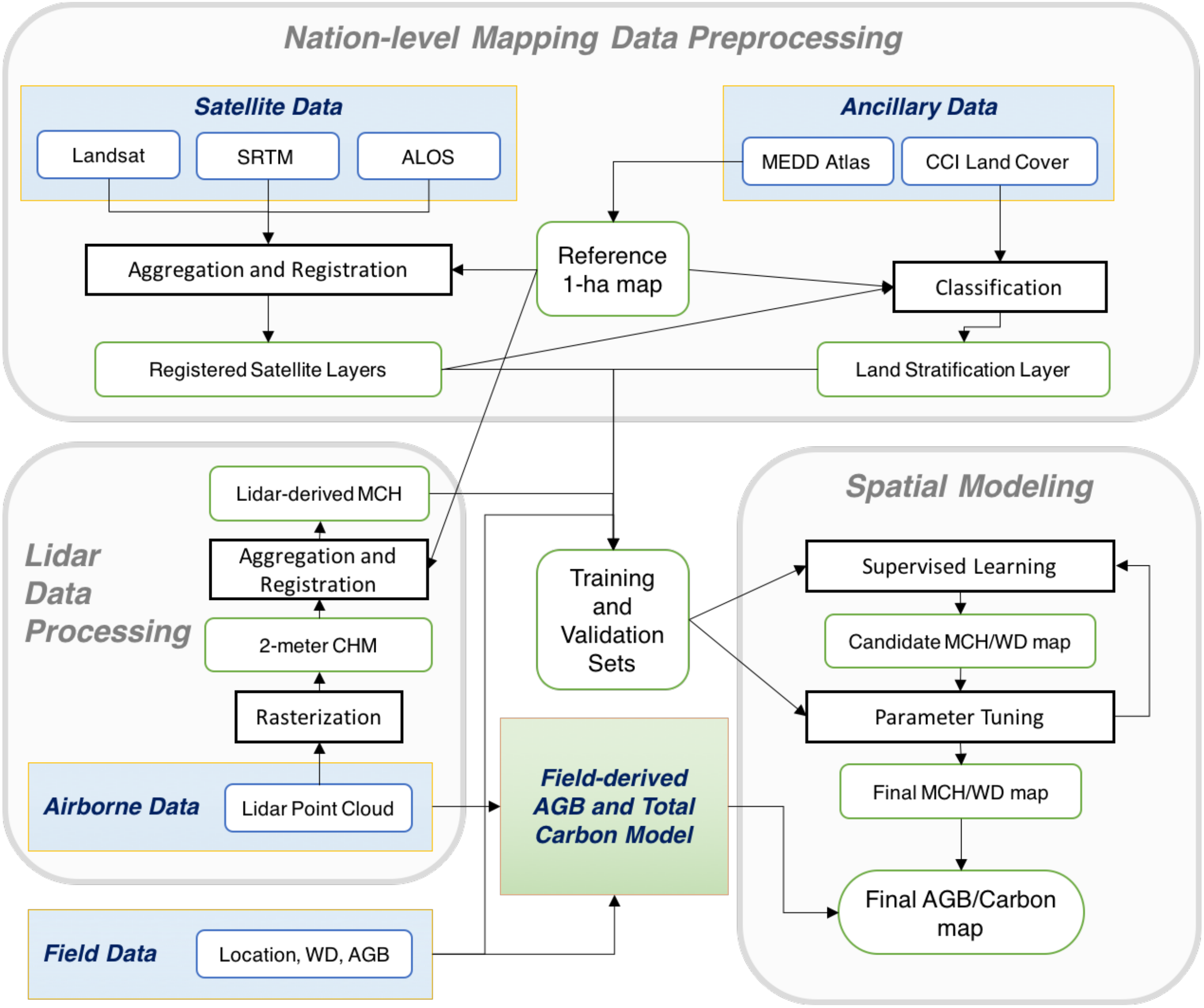

Supplementary Figure S2. Diagram of the national carbon mapping workflow.

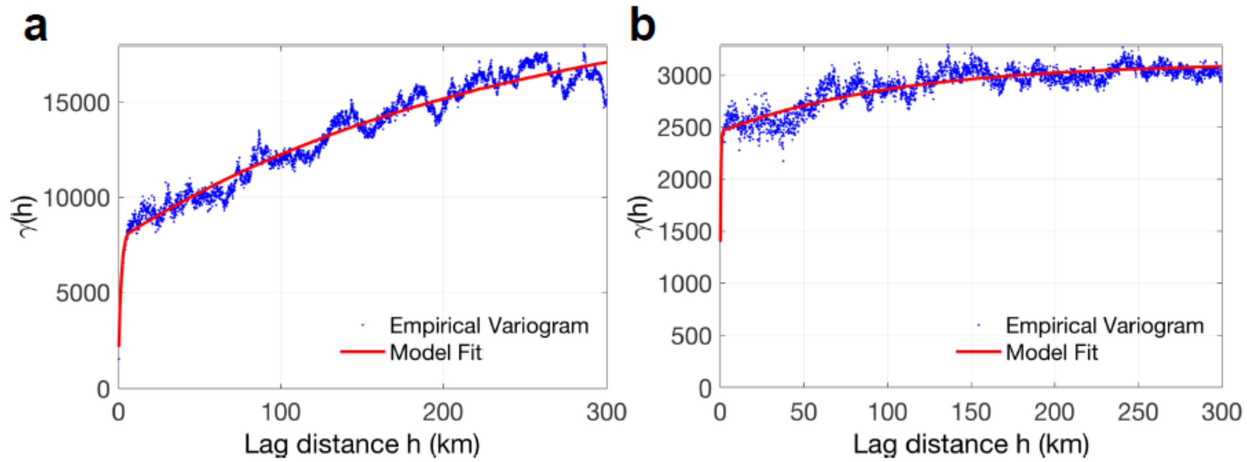

Supplementary Figure S3. Variogram plots showing spatial autocorrelation changing with the lag distance. (a) Variogram of original LiDAR-derived AGB values; (b) Variogram of the AGB prediction residual (the difference between predicted values and measurements).

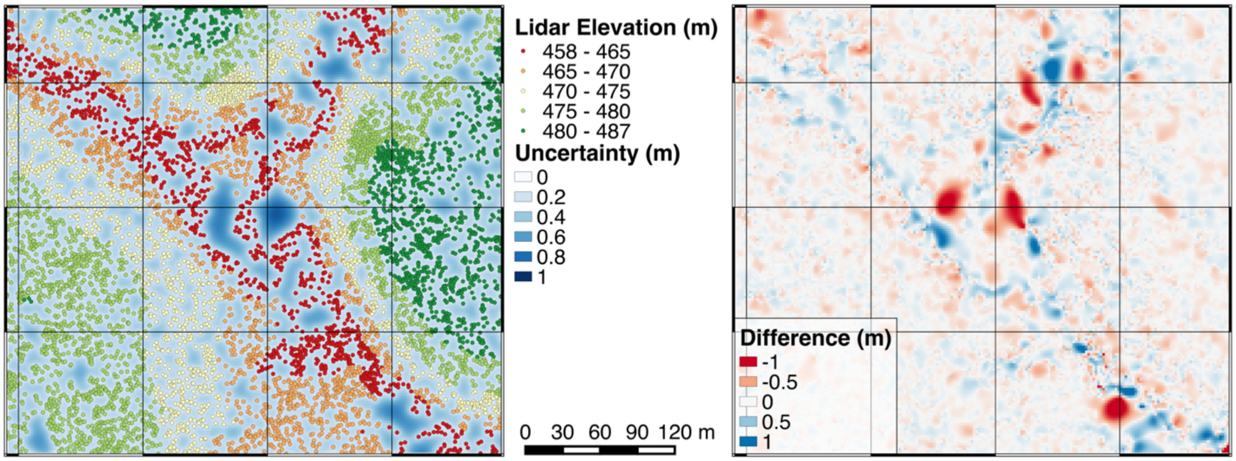

Supplementary Figure S4. Evaluation of uncertainty using kriging. The areas with no LiDAR observations have the most uncertainty (left panel). The difference between natural neighbor interpolation and kriging prediction (right panel) also shows the biggest differences in these regions.

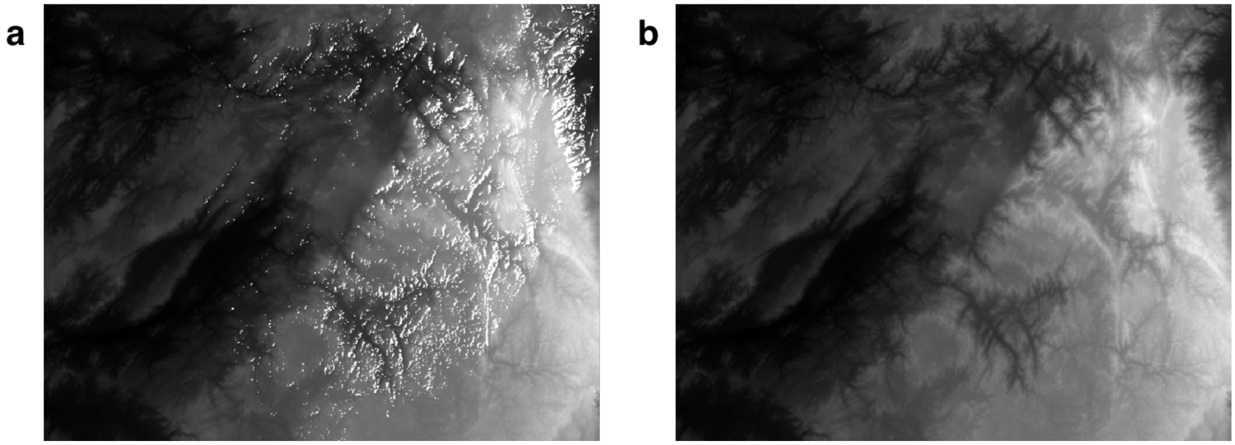

Supplementary Figure S5. Example of void-filling in SRTM. (a) the scattered white pixels are the void area with no valid observations in SRTM v3 data[38]. (b) We filled them using the ASTER GDEM v2 data[17]. The images were sampled from eastern DRC near the Mitumba Mountains and displayed using QGIS software[37].

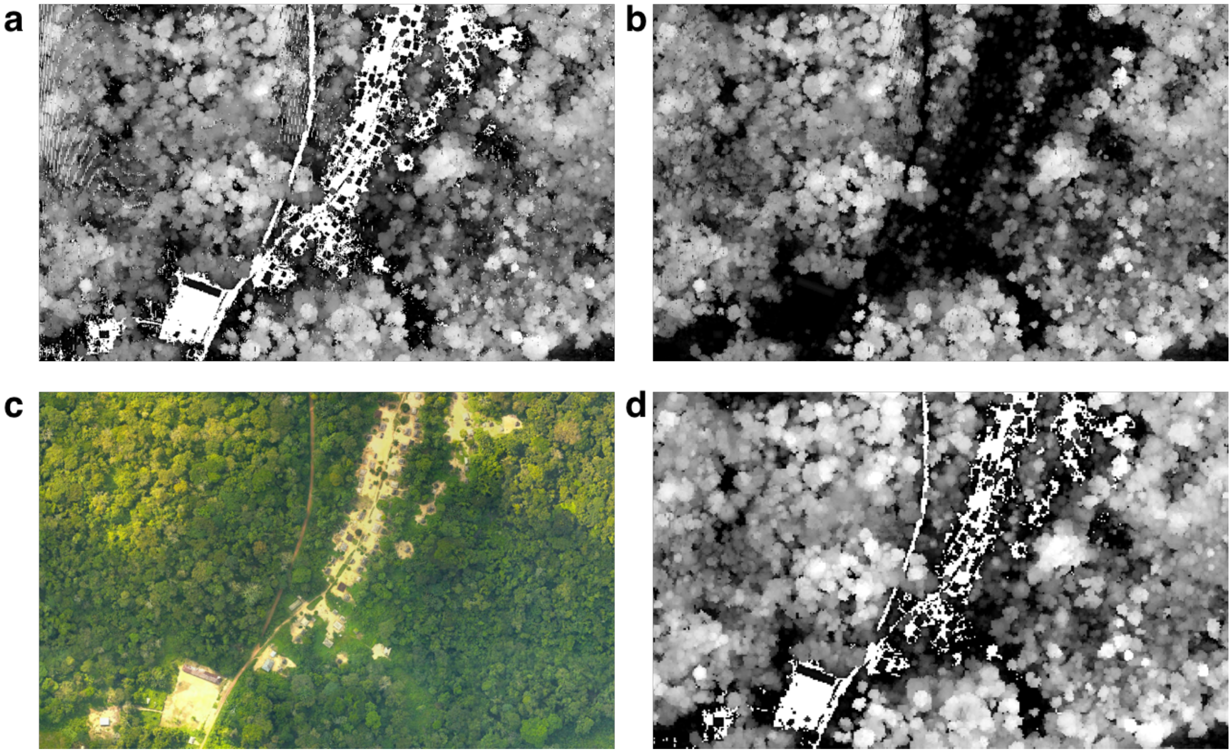

Supplementary Figure S6. Example images of data corrections. (a) 1-m CHM with no interpolation (data gaps shown in white); (b) 1-m CHM with interpolation (incorrect height values shown in black); (c) RGB orthophoto of the same location; (d) 2-m CHM with no interpolation (data gaps shown in white). Orthophotos were taken alongside LiDAR data in the same airborne campaign (see Supplementary Methods), and generated using GDAL[39] and QGIS[37] software.

Supplementary Table S1: Calibration plots (1ha) used to develop the LiDAR AGB model.

Source	Plot name	BA (m2/ha)	mean WD (g/cm3)	AGB (Mg/ha)	nb trees	MCH LiDAR
**Bastin**	41	26.64	0.68	321.85	341.00	29.19
**Bastin**	53	18.90	0.72	242.03	437.00	21.61
**Bastin**	123	26.40	0.70	349.91	386.00	26.95
**Bastin**	148	22.16	0.68	311.90	181.00	23.89
**Bastin**	151	25.40	0.64	320.30	279.00	25.49
**Bastin**	162	31.07	0.67	398.85	349.00	29.99
**Bastin**	166	26.31	0.65	293.39	350.00	29.38
**Bastin**	180	33.83	0.62	359.70	477.00	28.42
**Bastin**	184	29.10	0.66	395.88	385.00	32.42
**Bastin**	189	28.82	0.67	406.19	318.00	32.06
**Bastin**	199	29.65	0.68	457.83	378.00	33.04
**Bastin**	M2P2	26.76	0.71	309.42	476.00	24.86
**Bastin**	M2P1	24.47	0.67	291.57	447.00	25.61
**Bastin**	M2P9	19.12	0.59	203.32	139.00	25.22
**Bastin**	M2P13	21.00	0.63	182.52	380.00	22.20
**Bastin**	M2P12	19.09	0.62	217.09	110.00	23.95
**Bastin**	M2P11	21.55	0.61	191.67	426.00	20.79
**Bastin**	M2P10	24.03	0.68	247.55	356.00	24.85
**Bastin**	M2P14	8.19	0.59	29.53	378.00	15.18
**Bastin**	M2P15	14.57	0.50	121.13	415.00	19.63
**Bastin**	M2P16	17.35	0.62	160.40	265.00	20.23
**Yangambi**	Young regrowth-2	16.67	0.29	51.51	447.00	18.54
**Yangambi**	Fallow-1	5.24	0.30	12.20	350.00	11.67
**Yangambi**	Young regrowth-3	17.84	0.34	73.10	311.00	19.72
**Yangambi**	Fallow-2	2.01	0.31	4.06	132.00	13.24
**Yangambi**	Mixed-1	34.79	0.66	361.94	560.00	29.06
**Yangambi**	Mixed-5	26.93	0.63	286.47	329.00	29.35
**Yangambi**	Mono-dominant-1	31.80	0.71	401.29	342.00	32.83
**Yangambi**	Mono-dominant-2	32.14	0.72	374.54	435.00	29.04
**Yangambi**	Mono-dominant-3	30.58	0.70	365.68	376.00	31.10
**PARAP**	Parap-Gungu	19.11	0.54	151.43	518.00	11.33
**PARAP**	Parap-Mangai	26.97	0.61	405.93	406.00	29.58
**PARAP**	Parap-Mashambio	26.28	0.64	233.35	708.00	21.72
**PARAP**	Parap-SwaKibula	15.56	0.53	118.15	405.00	16.17
**PARAP***	Parap_Luhudi	17.10	0.52	124.01	552.00	14.69
**EQ DIAF**	07_01	24.29	0.49	194.20	275.00	21.99
**EQ DIAF**	08_02	33.08	0.57	361.37	517.00	22.30
**EQ DIAF**	11_05	24.07	0.79	309.22	521.00	23.86
**EQ DIAF**	12_06	25.04	0.66	270.83	333.00	28.22
**EQ DIAF**	13_07	15.89	0.69	214.98	193.00	27.81
**EQ DIAF**	14_08	23.25	0.68	342.46	368.00	21.65
**EQ DIAF**	15_09	25.48	0.57	235.26	404.00	19.25
**EQ DIAF**	16_10	27.49	0.58	348.97	181.00	28.56
**EQ DIAF**	17_11	8.52	0.66	68.80	196.00	14.75
**EQ DIAF**	SUP_I	18.32	0.79	241.20	385.00	15.38
**EQ DIAF**	SUP_II	31.27	0.54	281.18	352.00	25.27
**Ituri***	Edoro1	27.52	0.68	330.62	453.00	26.04
**Ituri***	Edoro1	26.00	0.72	331.16	477.00	28.06
**Ituri***	Edoro1	29.09	0.71	368.76	517.00	28.34
**Ituri***	Edoro1	30.33	0.71	404.74	456.00	28.64
**Ituri***	Edoro1	27.58	0.71	357.54	448.00	27.24
**Ituri***	Edoro1	28.31	0.71	344.42	449.00	29.52

**Ituri***	Edoro1	29.71	0.71	417.40	448.00	30.56
**Ituri***	Edoro1	31.75	0.71	408.22	487.00	29.91
**Ituri***	Lenda1	29.42	0.67	352.13	387.00	32.30
**Ituri***	Lenda1	34.29	0.68	486.40	355.00	34.30
**Ituri***	Lenda1	35.17	0.70	540.29	246.00	39.99
**Ituri***	Lenda1	34.99	0.70	514.88	270.00	37.22
**Ituri***	Lenda1	28.43	0.69	348.28	427.00	25.21
**Ituri***	Lenda1	31.17	0.69	412.84	408.00	33.88
**Ituri***	Lenda1	32.80	0.69	445.87	385.00	35.35
**Ituri***	Lenda1	32.32	0.70	477.73	298.00	36.34
**Ituri***	Lenda1	34.82	0.70	532.32	288.00	37.03
**Ituri***	Lenda2	34.88	0.67	467.39	441.00	32.74
**Ituri***	Lenda2	38.59	0.70	527.40	392.00	30.86
**Ituri***	Lenda2	29.89	0.68	410.24	330.00	34.06
**Ituri***	Lenda2	29.23	0.67	377.27	396.00	33.38
**Salonga**	Betamba	21.66	0.48	189.47	461.00	19.21
**Salonga**	Betamba	22.33	0.46	185.39	600.00	23.84
**Salonga**	Betamba	13.48	0.48	92.52	341.00	24.68
**Salonga**	Betamba	18.43	0.45	96.20	453.00	16.90
**Salonga**	Lokofa	24.25	0.56	199.84	490.00	18.83
**Salonga**	Lokofa	18.69	0.64	173.33	262.00	21.24
**Salonga**	Lokofa	20.75	0.61	208.00	228.00	19.78
**Salonga**	Lokofa	19.27	0.64	169.40	317.00	21.27
**Mai Ndombe**	15	21.32	0.69	262.81	241.00	24.56
**Mai Ndombe**	39	24.67	0.64	376.60	321.00	25.36
**Mai Ndombe**	80	24.72	0.69	322.89	209.00	29.04
**Mai Ndombe**	102	21.18	0.68	229.00	132.00	24.14
**Mai Ndombe**	289	30.56	0.70	390.79	459.00	26.11
**Mai Ndombe**	373	26.87	0.67	326.32	456.00	24.67
**Mai Ndombe**	408	30.67	0.66	282.93	599.00	19.16
**Mai Ndombe**	162	21.33	0.67	186.26	394.00	23.31
**Mai Ndombe**	227	26.79	0.60	251.22	404.00	28.25
**Mai Ndombe**	546	23.58	0.63	229.79	362.00	26.73
**Mai Ndombe**	627	18.29	0.66	150.72	376.00	18.61
**WWF**	24_6_KE_C_166	26.92	0.65	281.08	474.00	26.60
**WWF**	43_01_MA_L_152	20.28	0.64	244.49	305.00	23.49
**WWF**	44_02_MA_C_167	28.93	0.64	393.27	328.00	25.97
**WWF**	45_03_MA_C_178	22.86	0.51	192.89	343.00	21.33
**WWF**	MA_205	31.64	0.63	385.02	373.00	26.08
**WWF**	OR_87	29.77	0.59	370.48	348.00	27.07

* Sites where the 2nd equation of Eq. 2 was used to calculate AGB, due to the lack of tree height measurements.

Supplementary Table S2: CV results using Monte Carlo CV cases. CV1 and CV2 refer to different set ups of training and test sampling strategies (see Supplementary Methods).

CV Results	MSD	RMSE	R^2
CV1_1	1.42	60.76	0.78
CV1_2	-5.68	59.68	0.76
CV1_3	-3.48	60.87	0.74
CV1_4	-0.20	59.10	0.77
CV1_5	5.69	61.29	0.76
CV1_6	1.31	58.68	0.79
CV1_7	2.08	63.53	0.74
CV1_8	-1.15	60.25	0.75
CV1_9	1.70	61.13	0.75
CV1_10	2.35	61.05	0.74
CV2_1	-3.75	81.45	0.49
CV2_2	-24.88	72.99	0.59
CV2_3	-20.72	70.95	0.56
CV2_4	-11.38	62.16	0.65
CV2_5	-14.15	67.28	0.71
CV2_6	-13.63	74.22	0.72
CV2_7	8.04	71.34	0.74
CV2_8	19.13	69.78	0.78
CV2_9	16.59	65.45	0.78
CV2_10	0.14	64.92	0.69
**CV1 Mean**	**0.40±3.21**	**60.63±1.36**	**0.76±0.02**
**CV2 Mean**	**-4.46±15.22**	**70.05±5.54**	**0.67±0.10**

Supplementary Table S3: WorldClim variables, soil properties, and geographical features used in the Lasso regression for studying the DRC environmental controls. All values were aggregated into 0.25°x0.25° pixel resolution.

**Soil property**	**Description**	**Unit**
BIO1	Annual Mean Temperature	°C*10
BIO2	Mean Diurnal Range (max temp - min temp)	°C*10
BIO3	Isothermality (BIO2/BIO7) (* 100)	Unitless
BIO4	Temperature Seasonality (standard deviation *100)	Unitless
BIO5	Max Temperature of Warmest Month	°C*10
BIO6	Min Temperature of Coldest Month	°C*10
BIO7	Temperature Annual Range (BIO5-BIO6)	°C*10
BIO8	Mean Temperature of Wettest Quarter	°C*10
BIO9	Mean Temperature of Driest Quarter	°C*10
BIO10	Mean Temperature of Warmest Quarter	°C*10
BIO11	Mean Temperature of Coldest Quarter	°C*10
BIO12	Annual Precipitation	mm
BIO13	Precipitation of Wettest Month	mm
BIO14	Precipitation of Driest Month	mm
BIO15	Precipitation Seasonality (Coefficient of Variation)	Unitless
BIO16	Precipitation of Wettest Quarter	mm
BIO17	Precipitation of Driest Quarter	mm
BIO18	Precipitation of Warmest Quarter	mm
BIO19	Precipitation of Coldest Quarter	mm
CEC_T/CEC_S	Topsoil/Subsoil CEC in the soil	cmol kg^{-1}
OC_T/OC_S	Topsoil/Subsoil Organic Carbon	% weight
PH_T/PH_S	Topsoil/Subsoil PH (H2O)	Unitless
SLIT_T/SLIT_S	Topsoil/Subsoil Silt Fraction	%
CLAY_T/CLAY_S	Topsoil/Subsoil Clay Fraction	%
SAND_T/SAND_S	Topsoil/Subsoil Sand Fraction	%
Elevation Mean	Mean land elevation from SRTM	m
Elevation IQR	Interquartile range (25th to 75th percentiles) of land elevation from SRTM	m

Supplementary Table S4: Lasso regression results between DRC carbon density and environmental controls. The summary table selects the regularization parameter (λ) when keeping 6 out of 33 variables, and is sorted by the magnitude of parameter β. All values were aggregated into 0.25°x0.25° pixel resolution.

**Humid Forests**		**Miombo Woodlands**		**Swamp Forests**	
**Variables ()**	***β***	**Variables ()**	***β***	**Variables ()**	***β***
Mean T. of Driest Quarter (°C)	-0.408	P. Seasonality (Unitless)	-0.073	Mean Land Elevation (m)	1.588
Topsoil Organic Carbon (% weight)	-0.311	Annual Precipitation (mm)	0.037	Land Elevation Interquartile Range (m)	-0.787
Land Elevation Interquartile Range (m)	0.291	Subsoil Silt Fraction (%)	-0.035	Annual Mean Temperature (°C)	-0.164
P. Seasonality (Unitless)	-0.260	Mean Land Elevation (m)	-0.030	Min T. of Coldest Month (°C)	-0.141
Mean T. of Warmest Quarter (°C)	-0.154	Subsoil Organic Carbon (% weight)	0.017	Annual Precipitation (mm)	0.112
P. of Warmest Quarter (mm)	0.057	Topsoil Clay Fraction (%)	0.009	P. of Wettest Month (mm)	0.010
